# Octahedral oxide glass network in ambient pressure neodymium titanate

**DOI:** 10.1038/s41598-022-12342-x

**Published:** 2022-05-18

**Authors:** Stephen K. Wilke, Oliver L. G. Alderman, Chris J. Benmore, Jörg Neuefeind, Richard Weber

**Affiliations:** 1 Materials Development, Inc., Evanston, IL 60202 USA; 2 X-Ray Science Division, Advanced Photon Source, Argonne National Laboratory, Argonne, IL 60439 USA; 3 ISIS Neutron & Muon Source, Rutherford Appleton Laboratory, Chilton, Didcot, Oxon, OX11 0QX UK; 4 Neutron Science Division, Spallation Neutron Source, Oak Ridge National Laboratory, Oak Ridge, TN 37831 USA

**Keywords:** Structure of solids and liquids, Characterization and analytical techniques, Atomistic models

## Abstract

Rare-earth titanates form very fragile liquids that can be made into glasses with useful optical properties. We investigate the atomic structure of 83TiO_2_-17Nd_2_O_3_ glass using pair distribution function (PDF) analysis of X-ray and neutron diffraction with double isotope substitutions for both Ti and Nd. Six total structure factors are analyzed (5 neutron + 1 X-ray) to obtain complementary sensitivities to O and Ti/Nd scattering, and an empirical potential structure refinement (EPSR) provides a structural model consistent with the experimental measurements. Glass density is estimated as 4.72(13) g cm^−3^, consistent with direct measurements. The EPSR model indicates nearest neighbor interactions for Ti-O at $\overline{r}_{TiO}$ = 1.984(11) Å with coordination of $n_{TiO}$ = 5.72(6) and for Nd-O at $\overline{r}_{NdO}$ = 2.598(22) Å with coordination of $n_{NdO}$ = 7.70(26), in reasonable agreement with neutron first order difference functions for Ti and Nd. The titanate glass network comprises a mixture of distorted Ti-O_5_ and Ti-O_6_ polyhedra connected via 71% corner-sharing and 23% edge-sharing. The O-Ti coordination environments include 15% nonbridging O-Ti_1_, 51% bridging O-Ti_2_, and 32% tricluster O-Ti_3_. This structure is highly unusual for oxide glasses melt-quenched at ambient pressure, as it consists of Ti-O_x_ predominantly in octahedral (with nearly no tetrahedral) coordination.

## Introduction

Titanate network glasses are formed from very fragile liquids that would not typically be expected to vitrify. Lanthanum and neodymium titanates were the first to be successfully vitrified, using roller quenching^1^, with compositions near deep eutectics in the binary oxide phase diagrams. Application of containerless melting later led to discovery of a larger family of titanate glasses^2–4^. These materials are promising for optical technologies because of their high refractive indices (> 2.1^1,5^), low dispersion^3^, wide transmission range over visible and infrared wavelengths^1,3^, and ferroelectric and dielectric properties as glass-ceramics^6–8^. Many titanate glasses also contain large fractions of rare-earth oxides (11–20 mol.% RE_2_O_3_), making them technologically interesting because of the optical activity of ions such as Nd^3+^, Pr^3+^, and Er^3+^^4^. Optimization of these glasses’ attractive properties can benefit from detailed knowledge of their structure-processing-property relationships, which motivates studies of their atomic structure and glass formation. For example, the relative populations of Ti-O_5_ and Ti-O_6_ species in La_4_Ti_9_O_24_ glass have been found to play an important role in determining electric susceptibility and refractive index^5^. More generally, rare-earth titanate glasses contain no canonical glass former (e.g., SiO_2_, P_2_O_5_, B_2_O_3_, GeO_2_), which stimulates curiosity about the structure underlying their glass forming behavior. One important question is to what extent O-Ti_3_ species can be topologically accepted before titanate glass formation is no longer possible, which may explain the compositional limits for rare-earth titanate glasses^4^.

Arai et al*.* published the first study of atomic structure in a rare-earth titanate glass, La_4_Ti_9_O_24_, using high energy X-ray diffraction (HEXRD) and neutron diffraction (ND)^5^. These total scattering methods and the related pair distribution function (PDF) analysis are powerful tools for probing atomic structure in glassy materials^9,10^. However, analysis of a total PDF is often insufficient to reliably calculate atomic coordination in binary oxides. For a binary oxide, the three comprising elements (two cations and oxygen) result in six distinct atomic partial pair correlations. The total PDF that is obtained from a single scattering measurement represents a weighted summation of these six partial pair correlations, and typically the cation-oxygen pair distances (and perhaps others) overlap in real space, making it difficult to accurately separate them during PDF analysis. This challenge was partially addressed by Arai et al*.* using a HEXRD/ND difference function to eliminate the O-O partial for La_4_Ti_9_O_24_^5^. Still, substantial overlap of the Ti-O and La-O correlations required fitting of multiple Gaussian functions, from which separate coordination numbers were calculated for the two cations. Similar challenges of PDF peak overlap were encountered by Maruyama et al*.* in studying Sm_4_Ti_9_O_24_ glass^11^ and by Alderman et al*.* in a study of rare-earth titanate melts and glasses^4^. To address peak overlap, Maruyama et al*.* used ND with isotopic substitution for Ti and Sm, which provided neutron difference functions that mostly isolated the first peaks for Ti-O, Sm-O, and O-O partial pair correlations. Alderman et al*.*, focusing primarily on 83TiO_2_-17La_2_O_3_ glass, augmented the determination of Ti-O coordination with Ti X-ray absorption near-edge structure (XANES) measurements, but the La-O coordination remained difficult to determine confidently from the PDF because of overlap with O-O and cation-cation partials.

Here, we build upon these past studies using ND with double isotope substitution, HEXRD, and complementary structural modeling for 83TiO_2_-17Nd_2_O_3_ glass. Similar to Maruyama et al*.*’s work with Sm_4_Ti_9_O_24_ (18.2 mol.% Sm_2_O_3_)^11^, the ND difference functions are a powerful analysis technique for 83TiO_2_-17Nd_2_O_3_ because of the large differences in coherent neutron scattering lengths for isotopes of both Ti and Nd. The large difference in Nd isotopes’ scattering lengths has been used previously to isolate the Nd-O partial in Nd-doped silicate and aluminate glasses^12^, and Nd isotopes do not present as problematic of thermal neutron resonances as those for Sm isotopes^11^. In comparison to the rare-earth titanate glass studies by Arai et al*.*^5^ (1 ND + HEXRD), Maruyama et al.^11^ (3 ND), we present 6 total measurements for neodymium titanate (5 ND + HEXRD).

In this work, we describe the preparation of 83TiO_2_-17Nd_2_O_3_ glass beads, using aerodynamic levitation and laser heating, to obtain six different combinations of Ti and Nd isotopic substitutions. Atomic structure is probed using ND and HEXRD. Glass density is estimated from the HEXRD PDF, which is compared with gas pycnometry and densities for similar glasses found in the literature. Despite obtaining six diffraction measurements, each with unique weightings of the six atomic partial pair correlations, direct determination of all partials remains prone to high uncertainties for some atomic pairs. Thus, to augment the experimental measurements, the glass structure is modeled using empirical potential structure refinement (EPSR) to the data, from which all six partials are estimated and further compared against the ND first order difference functions for Ti and Nd. EPSR effectively constrains the matrix inversion required to obtain the partial pair correlations from the diffraction data to a structural model consistent with a realistic three-dimensional distribution of atoms at the known glass composition and bulk density. The structural model is then used to analyze the atomic coordination environments and glass network structure.

## Results and discussion

### Glass formation and composition

Glass forming ability of neodymium titanate was first demonstrated by Kozuka et al*.* using twin-roller quenching^1^ and then by Alderman et al*.* using aerodynamic levitation^4^. In general, rare-earth titanates are challenging to vitrify, as evidenced by the narrow compositional range over which they form glasses (e.g., 15–20 mol.% Nd_2_O_3_)^1,4^ and the large cooling rates required to avoid crystal nucleation, even in containerless conditions^13,14^. Here, glasses of 83TiO_2_-17Nd_2_O_3_ were prepared using aerodynamic levitation and laser heating^15^, a containerless processing method effective for vitrifying reluctant glass formers, since heterogeneous crystal nucleation at solid container walls or by container dissolution impurities is avoided^13^.

**Figure 1:**
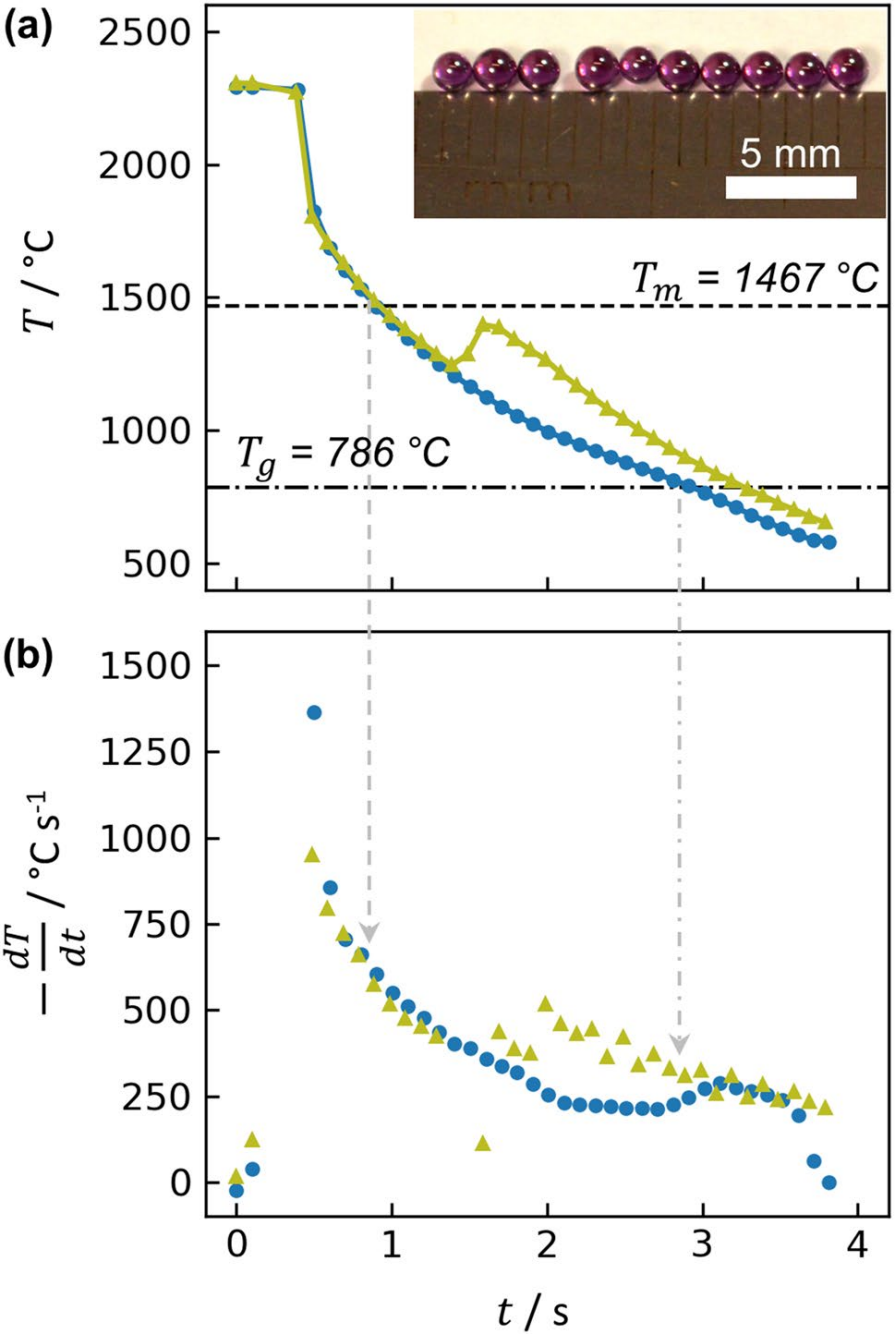
(**a**) Temperature and (**b**) cooling rates of two exemplary beads of 83TiO_2_-17Nd_2_O_3_ prepared via aerodynamic levitation and laser melting. For sufficiently fast cooling, beads formed glass (blue curve); otherwise, recalescence occurred due to crystallization and caused a momentary increase in temperature (green curve). Inset: photo of glass beads.

For glass synthesis, a bead of 83TiO_2_-17Nd_2_O_3_ was levitated and melted ($T_{m}$ = 1467°C^16^), and then the laser was turned off to achieve rapid cooling. Two examples of beads’ cooling behavior are shown in Fig. 1. As the levitated liquid bead cools, the supercooled liquid may cool below $T_{g}$ (786°C^1^) to form a glass (Fig. 1a, blue curve), or recalescence may occur before the droplet reaches $T_{g}$ (Fig. 1a, green curve), signifying latent heat release during crystallization, which momentarily raises the sample temperature to near $T_{m}$. Crystallization from the supercooled liquid was often observed if the cooling rate was not sufficiently large, which necessitated the use of small beads no larger than ~ 11 mg or ~ 1.6 mm in diameter. For these bead sizes, the cooling rate (Fig. 1b) was typically > 1000 °C s^−1^ above 1800 °C and then declined as the bead cooled and radiative contributions to heat transfer lessened. Cooling rates were ~ 600 °C s^−1^ near $T_{m}$ and ~ 250 °C s^−1^ near $T_{g}$ (Fig. 1, gray vertical arrows). Glass beads were transparent with a strong purple hue and, in most cases, free of voids or bubbles (Fig. 1a, inset).

For HEXRD, glass beads were prepared from TiO_2_ and Nd_2_O_3_ powders with natural isotopic abundance. For ND, glass beads were prepared for five different isotopic substitutions: ^46^Ti-^nat^Nd, ^48^Ti-^nat^Nd, ^nat^Ti-^144^Nd, ^nat^Ti-^145^Nd, and ^null^Ti-^nat^Nd, where “nat” refers to natural isotopic abundance and ‘null’ refers to an isotopic composition tuned such that the scattering length is zero, on average. Due to cost and supply limitations on isotope-enriched powders, only small batches of the isotopically-unique samples were prepared, each ranging 62–114 mg or, equivalently, 6–10 beads. To confirm sample composition, energy dispersive X-ray spectroscopy measurements were collected across 10 sites of the polished cross-section for a ^nat^Ti-^nat^Nd bead. The mean and standard deviation of composition were 18.2(1) mol.% Nd_2_O_3_, slightly richer in Nd_2_O_3_ than targeted, which may be caused by slight TiO_2_ evaporation during laser melting or powder weighing errors. The bead cross-section appeared homogeneous. Compositional uniformity and absence of crystallinity across the different isotopically-enriched samples were confirmed by consistency of the PDFs obtained from HEXRD (Fig. S1).

### Density

Glass density was estimated from the HEXRD PDFs of all samples. In the nonphysical region ($r$ < 1.2 Å), the absence of any atomic bonds implies that the total PDF, $T\left( r \right)$, should be zero (assuming the free atom approximation). Equivalently, for the differential PDF, $D\left( r \right) \to - 4\pi \rho r$ (see Eqns. 12–13 in Methods, where PDFs are mathematically defined)^10,17^. This limiting behavior of $D\left( r \right)$ at low-$r$ provides a route to estimate density. However, oscillations do appear in the nonphysical region due to two effects: (i) truncation artifacts resulting from a finite $Q$ range of the measurement ($Q_{max}$ in Eq. 12), and (ii) residual imperfections from corrections to the structure factor. The effect of (ii) is minimized using the “top hat” convolution^18^ described in the Methods. To minimize the effect of (i) on density estimation, the first two peaks in the PDF were fitted with Gaussian distributions convolved with their associated peak functions, including truncation effects. These fitted peaks and truncation oscillations were subtracted from $D\left( r \right)$, and the sample density was determined by minimizing the sum-square difference between $D\left( r \right)$ (peaks subtracted) and $- 4\pi \rho r$ over the range of $r$ = 0–1.4 Å (Fig. S2).

For the six samples, the weighted mean and standard deviation of density are $\rho$ = 4.72(13) g cm^−3^ or, equivalently, 0.07689 atoms Å^−3^. Density was also calculated from helium pycnometry (Table S1), yielding $\rho$ = 4.55(39) g cm^−3^, but the uncertainty for pycnometry was larger than for HEXRD due to the very small sample volumes. Thus, the HEXRD value is used for all analyses here. For comparison, the density of the compositionally closest crystalline phase, Nd_4_Ti_9_O_24_ (18.2 mol.% Nd_2_O_3_), is 5.178 g cm^−3^^19^. The most pertinent glass estimates from literature are for the compositional analogue 83TiO_2_-17La_2_O_3_, for which Alderman et al*.*^4^ used 4.84 g cm^−3^_,_ based on interpolation of Arai et al*.*’s Archimedes method measurements of La_4_Ti_9_O_24_ and La_4_Ti_11_O_28_ glasses^5^. Assuming the same molar volume as 83TiO_2_-17La_2_O_3_, this would predict 4.91 g cm^−3^ for 83TiO_2_-17Nd_2_O_3_, which is 3.9% larger than the density estimated from HEXRD. Maruyama et al*.* reported a density of 5.42 g cm^−3^ for Sm_4_Ti_9_O_24_ glass^11^ (10.4% larger than the 83TiO_2_-17Nd_2_O_3_ HEXRD estimate), though without mention of the measurement technique or details, so a critical comparison with the results is not possible here.

### X-ray and neutron diffraction

The five unique combinations of Ti and Nd isotope substitutions were chosen to provide a set of ND measurements with large differences in coherent neutron scattering lengths, $b$, and, consequently, the Faber-Ziman weighting factors that determine how much each atomic partial pair correlation contributes to the total PDF^10,20,21^. With these five ND measurements plus one HEXRD measurement, it should be possible to empirically determine the entire set of six partials. ND samples using ^nat^Nd with either ^46^Ti ($b$ = 4.93(6) fm) or ^48^Ti ($b$ =  − 6.08(2) fm)^22^ provide a robust Ti first order difference function, and samples using ^nat^Ti with either ^144^Nd ($b$ = 2.8(3) fm) or ^145^Nd ($b$ = 14(2) fm)^22^ provide a clearly resolved Nd-O peak in the Nd first order difference. The ^null^Ti sample was included to obtain a Ti second order difference function, and the HEXRD measurement provides data with larger weighting to the cations than is present in ND. It is important to note the large uncertainty in neutron scattering length for ^145^Nd, which is discussed in the Supplementary Information (SI) along with other sources of uncertainty.

**Figure 2:**
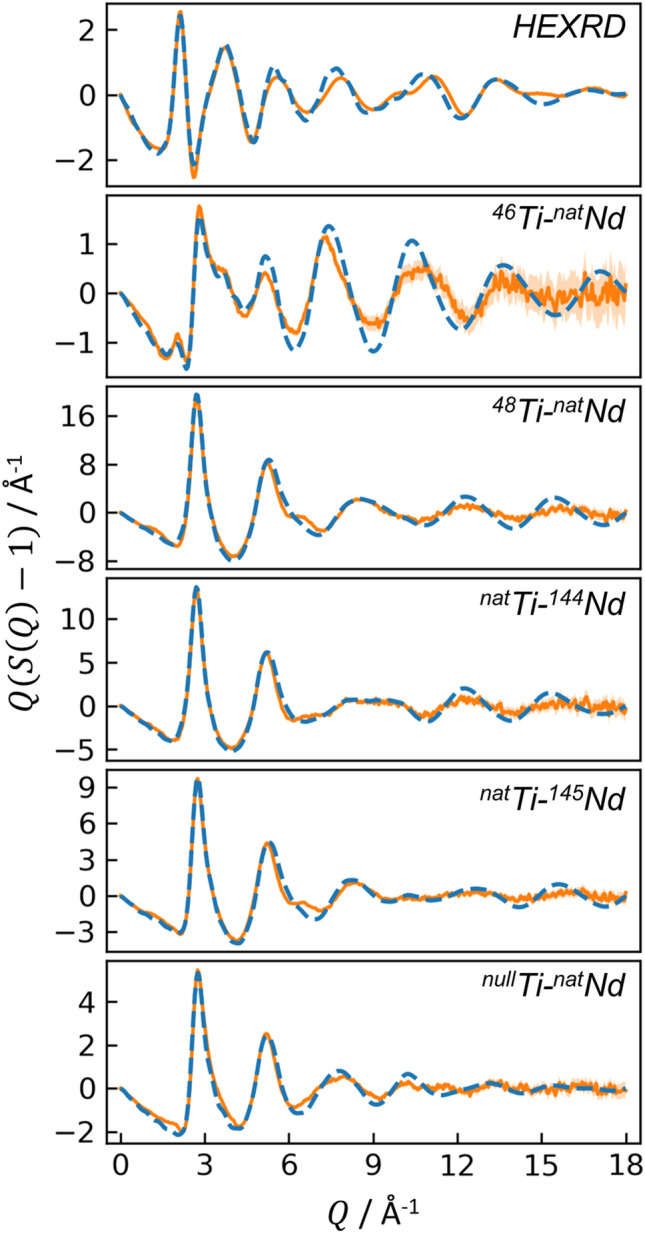
Interference functions for the HEXRD and five ND measurements. Solid orange curves are the experimental data, with uncertainty shown by light orange shading; dashed blue curves are the EPSR results.

The six measured interference functions, $Q\left( {S\left( Q \right) - 1} \right)$, are given in Fig. 2. The magnitude of the structure factors, $S\left( Q \right)$, varies between samples because they have been normalized by the average scattering lengths squared (Eq. 10 and Eq. 15 in Methods): $<b>^{2}$ for ND, or $<f\left( Q \right)>^{2}$ for HEXRD. Elemental and average scattering lengths for each sample are provided in Table S2. The first peak in $S\left( Q \right)$ is located at $Q_{1}$ = 2.13 Å^−1^ for HEXRD and 2.67–2.79 Å^−1^ for ND (except for ^46^Ti-^nat^Nd, which exhibits a small peak around 2.13 Å^−1^, similar to HEXRD). These are principal peaks, rather than first sharp diffraction peaks, based on Price et al*.*’s definition ($Q_{1} r_{1} \cong$ 2.5)^23^, which indicates a lack of intermediate range order in the neodymium titanate glass network.

**Figure 3:**
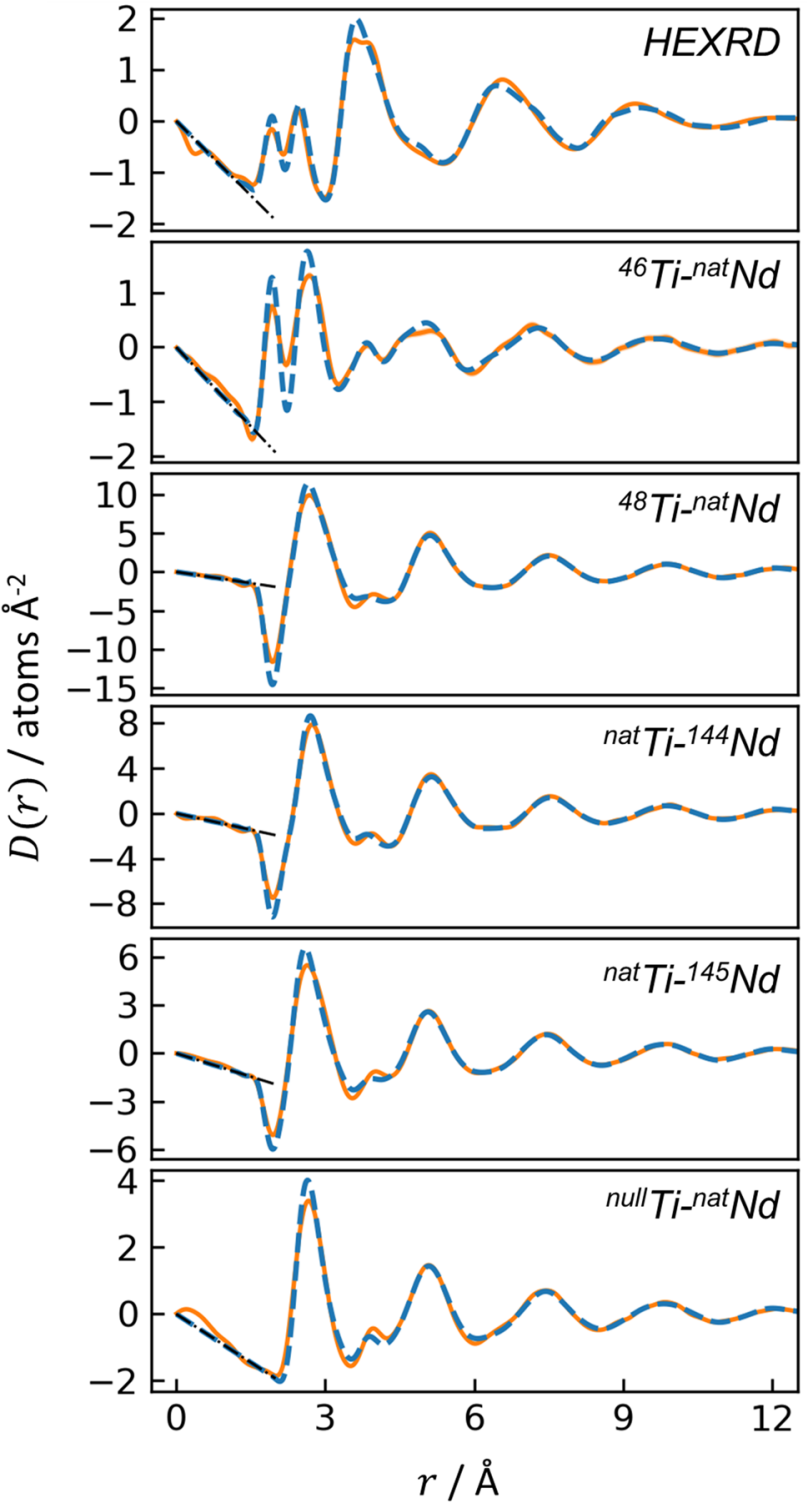
Differential PDFs for the HEXRD and five ND measurements. Solid orange curves are the experimental data, for which the uncertainty arising from diffraction measurements is imperceptibly small; dashed blue curves are the EPSR simulation. Dashed black lines indicate the initial slope given by $- 4\pi \rho$.

The differential PDFs, $D\left( r \right)$, are provided in Fig. 3. A few features immediately distinguish the different measurements. The first peak near 2.0 Å, corresponding to Ti-O, is absent in the ^null^Ti-^nat^Nd ND sample and appears negative for ND samples containing ^48^Ti or ^nat^Ti, which both have negative scattering lengths. The greater sensitivity of HEXRD to heavy elements is evident by the large, broad peak at ~ 3.7 Å corresponding to cation-cation pairs, which are less prominent in the ND measurements. The initial slopes of $D\left( r \right)$ are in good agreement with $- 4\pi \rho$^10^ (Fig. 3, dashed black lines), indicating proper normalization of the data. ND normalization was particularly challenging given the small samples (62–114 mg per measurement), as described in the SI. The uncertainty in $S\left( Q \right)$ was propagated in the Fourier transform to $D\left( r \right)$ with the method used by Skinner et al*.*^24^ and Weitkamp et al*.*^25^, but the resulting uncertainty in the PDF is so small that it is imperceptible in Fig. 3.

### Atomic partial pair correlations from diffraction

Analysis of partial pair correlations and atomic coordination is approached via three strategies: ND first order differences, a direct matrix inversion solution for all six partial structure factors, and EPSR. Direct peak fitting to the total PDFs was not performed due to substantial overlap of the Ti-O, Nd-O, and O-O partials. Results of total PDF fitting have been reported for 83TiO_2_-17La_2_O_3_ glass^4^, but since Nd has a smaller ionic radius than La (0.983 vs. 1.032 Å), peak overlap of Ti-O with Nd-O is more severe than with La-O.

**Figure 4:**
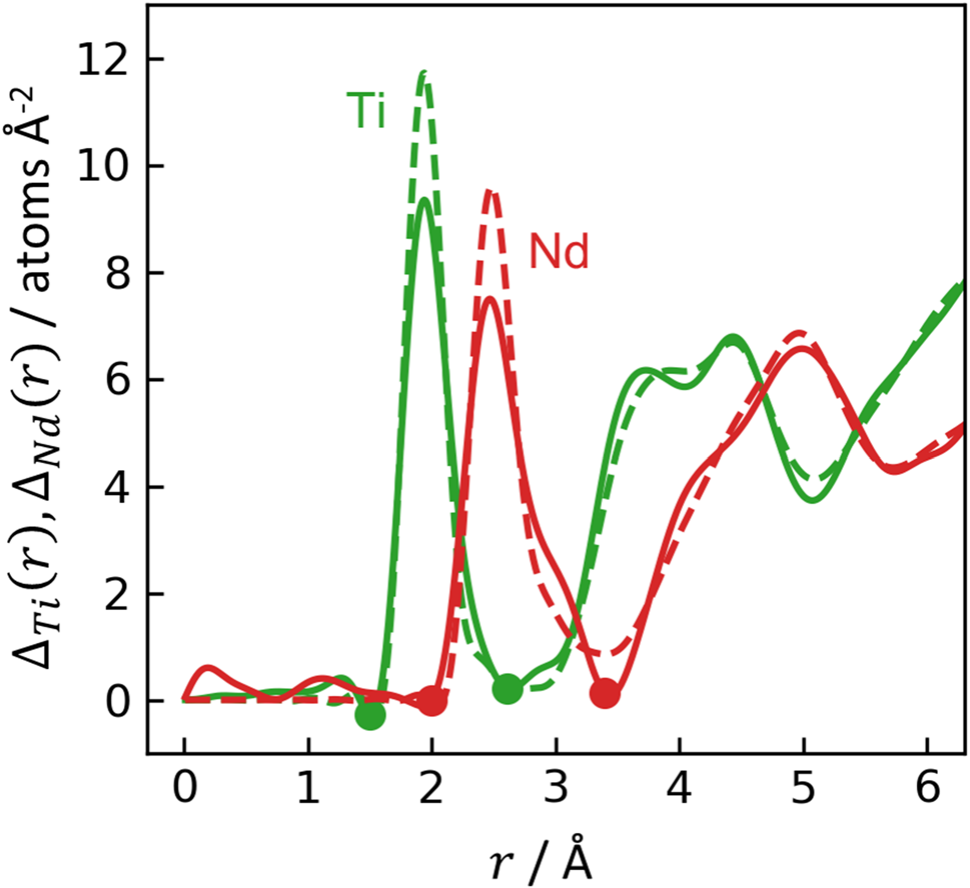
First order difference functions for Ti (green) and Nd (red) after dividing by the appropriate weighting factors for the first peaks to correspond to unweighted Ti-O and Nd-O partial pair correlations. The integration limits used to extract coordination numbers are marked with colored dots. Experimental and EPSR results are shown with solid and dashed curves, respectively.

First order difference functions exploit the difference in neutron scattering from samples that are identical except for the substitution of one isotope. The samples for this study were chosen to obtain separate first order differences for Ti (^46^Ti-^nat^Nd and ^48^Ti-^nat^Nd) and Nd (^nat^Ti-^144^Nd and ^nat^Ti-^145^Nd). Figure 4 (solid curves) shows the functions $\Delta_{Ti} \left( r \right)$ and $\Delta_{Nd} \left( r \right)$, which are the first order differences after division by the appropriate weighting factors so that their first peaks correspond to the unweighted partial pair correlations, $t_{TiO}$ and $t_{NdO}$. Integration of these peaks gives coordination numbers of $n_{TiO}$ = 5.43(15) and $n_{NdO}$ = 7.95(35). (Full procedures for obtaining difference functions, coordination numbers, and estimating uncertainty are provided in the SI.) Based on a comparison of $\Delta_{Ti} \left( r \right)$ and $\Delta_{Nd} \left( r \right)$, the Ti-Ti correlation likely forms the peak near 3.75 Å present in $\Delta_{Ti} \left( r \right)$ but absent in $\Delta_{Nd} \left( r \right)$, and the Ti-Nd and Nd-Nd correlations contribute to the overlapping peaks in $\Delta_{Nd} \left( r \right)$ beginning around 4.17 Å.

Each sample’s structure factor (Eq. 10 and Eq. 15) can be expressed as a summation of the atomic-pair partial structure factors, $s_{ij}$:

1 $$S\left( Q \right) = \mathop \sum \limits_{i,j \ge i} W_{ij} s_{ij}$$

where $W_{ij}$ are the normalized Faber-Ziman weighting factors^20^,

2 $$W_{ij} = \frac{{\left( {2 - \delta_{ij} } \right)c_{i} c_{j} f_{i} \left( Q \right)f_{j} \left( Q \right)}}{{<f\left( Q \right)>^{2} }}$$

and the summation in Eq. 1 is over all distinct atomic pairs (i.e., Ti-Ti, Ti-Nd, Ti-O, Nd-Nd, Nd-O, and O-O). Equation 2 defines the weighting factors for HEXRD measurements in terms of the $Q$-dependent X-ray atomic form factor, $f_{i} \left( Q \right)$. The same expression is applied for ND by replacing $f_{i} \left( Q \right)$ with the coherent neutron scattering length, $b_{i}$. Values for $c_{i}$, $W_{ij}$, and $<f\left( Q \right)>^{2}$ or $<b>^{2}$ of each sample are given in Table S2. The set of six measurements can then be expressed by a matrix equation^26,27^,

3 $$\left[ {\begin{array}{*{20}c} {S_{1} } \\ {\begin{array}{*{20}c} {S_{2} } \\ {\begin{array}{*{20}c} {S_{3} } \\ {S_{4} } \\ \end{array} } \\ {S_{5} } \\ {S_{6} }\\ \end{array} } \\ \end{array} } \right] = \left[ {\begin{array}{*{20}l} {W_{1,TiTi} } \hfill & {W_{1,TiNd} } \hfill & {W_{1,TiO} } \hfill & {W_{1,NdNd} } \hfill & {W_{1,NdO} } \hfill & {W_{1,OO} } \hfill \\ {W_{2,TiTi} } \hfill & {W_{2,TiNd} } \hfill & {W_{2,TiO} } \hfill & {W_{2,NdNd} } \hfill & {W_{2,NdO} } \hfill & {W_{2,OO} } \hfill \\ {W_{3,TiTi} } \hfill & {W_{3,TiNd} } \hfill & {W_{3,TiO} } \hfill & {W_{3,NdNd} } \hfill & {W_{3,NdO} } \hfill & {W_{3,OO} } \hfill \\ {W_{4,TiTi} } \hfill & {W_{4,TiNd} } \hfill & {W_{4,TiO} } \hfill & {W_{4,NdNd} } \hfill & {W_{4,NdO} } \hfill & {W_{4,OO} } \hfill \\ {W_{5,TiTi} } \hfill & {W_{5,TiNd} } \hfill & {W_{5,TiO} } \hfill & {W_{5,NdNd} } \hfill & {W_{5,NdO} } \hfill & {W_{5,OO} } \hfill \\ {W_{6,TiTi} } \hfill & {W_{6,TiNd} } \hfill & {W_{6,TiO} } \hfill & {W_{6,NdNd} } \hfill & {W_{6,NdO} } \hfill & {W_{6,OO} } \hfill \\ \end{array} } \right]\left[ {\begin{array}{*{20}c} {\begin{array}{*{20}c} {s_{TiTi} } \\ {s_{TiNd} } \\ \end{array} } \\ {\begin{array}{*{20}c} {\begin{array}{*{20}c} {s_{TiO} } \\ {s_{NdNd} } \\ \end{array} } \\ {s_{NdO} } \\ {s_{OO} } \\ \end{array} } \\ \end{array} } \right]$$

where $S_{1}$–$S_{5}$ are ND measurements and $S_{6}$ is HEXRD, for which the weighting factors are $Q$-dependent. Inversion of the weighting factor matrix (Eqn. S16 in the SI) provides a direct, empirical solution for the six partial structure factors.

However, the partial pair correlations obtained from the matrix solution are not equally reliable. The atomic pairs with large weighting factors in several of the measurements are likely to be accurately determined from the matrix solution, but atomic pairs with small weighting factors across all measurements are not expected to be reliably determined. To illustrate this point, it is helpful to compare the magnitude of the $W_{ij}$ between samples. For samples with negative scattering lengths, it is not intuitive to directly compare values of $W_{ij}$, so a modified version of the weighting factors is defined that directly represents the percentage weighting of each atomic pair to the total PDF:

4 $$W_{ij}^{\prime } = 100\left| {\frac{{W_{ij} }}{{\mathop \sum \nolimits_{i,j \ge i} \left| {W_{ij} } \right|}}} \right|$$

Based on the mean values of $W_{ij}^{\prime }$ (Table S2), three atomic pairs are strongly weighted in the matrix: O-O (43.3%), Nd-O (20.6%), and Ti-O (20.2%). The remaining three atomic pairs are weakly weighted and thus are not expected to be reliably determined by the matrix solution: Ti-Nd (7.4%), Ti-Ti (4.2%), and Nd-Nd (4.2%).

An alternate evaluation of the matrix solution robustness is the matrix determinant, which indicates solution stability, with values near 0 reflecting an ill-conditioned problem and values near ± 1 indicating a robust solution. Due to the relatively small differences in weighting factors for most ND isotope substitution measurements, absolute values of the determinant typically range from 0.0021 to 0.03^27–30^, which in most cases were still used to justify the obtained partial pair correlations. For the current set of samples, the matrix determinant is − 0.00023, roughly an order of magnitude smaller than prior studies. (It is worth noting that the examples of prior work are all studies of binary compounds, for which the matrix solution is for three partial structure factors, in contrast to the ternary compound here, for which the solution requires six partial structure factors.)

Still, a matrix solution was explored. The resulting partial structure factors and partial PDFs are shown in Figs. S3 and S4, respectively, with comparison to the partials obtained via EPSR. Due to the large uncertainty in some $s_{ij}$, propagated from the sample structure factors (Fig. 2), a $Q_{max}$ of 12 Å^−1^ was used to obtain the partial PDFs in Fig. S4 (for partials from both the matrix solution and from EPSR). The O-O partial shows the best agreement between the matrix inversion solution and EPSR, which correlates with the large O-O average weighting among all measurements. The Nd-O and Ti-O partials show fair agreement between the matrix solution and EPSR, as expected given their moderate weighting among all measurements. As anticipated, the matrix solution partials that are poorly-weighted in the measurements (Nd-Nd, Nd-Ti, Ti-Ti) do not have good agreement with EPSR.

The second order neutron difference function for Ti is identical to the Ti-Ti partial given in Figs. S3 and S4 (see Eqn. S16). The location of its PDF first peak, circa 3.3 Å, is in general agreement with EPSR, though the peak shapes differ. The EPSR Ti-Ti partial exhibits two overlapping contributions to the first peak, ~ 2.9 and ~ 3.6 Å, which correspond to edge- and corner-sharing of Ti-O_x_ polyhedra (discussed later).

### Structural model

EPSR offers a final, alternate approach to the partial pair correlations. The EPSR simulation provides a structural model with charge ordering imposed by reasonable pairwise Coulombic and dispersive interactions, minimally perturbed by empirical terms to obtain agreement with scattering data^21,31^. The EPSR model is constrained to an actual three-dimensional distribution of atoms with the measured (from HEXRD) bulk density. Because EPSR uses all six diffraction measurements as constraints for the model, and because it thoroughly samples the possible structural configurations of the system, it is likely the most reliable means of obtaining partials.

The interference functions and PDFs from EPSR are shown in Figs. 2 and 3, respectively, with dashed blue lines. In general, there is good agreement between experiment and EPSR, with two areas of exception. First, for all samples, EPSR predicts taller, narrower Ti-O peaks than observed experimentally, though the average Ti-O coordination from EPSR vs. experiment are still similar. Overestimation of Ti-O peak height is an issue observed previously for an EPSR model of molten BaTiO_3_^21^. Second, for the ^46^Ti-^nat^Nd sample, the height and integral of the EPSR-predicted Nd-O peak is larger than in the experimental data. The EPSR-predicted ND first order difference functions are shown in Fig. 4 (dashed curves), which again are in good agreement with HEXRD/ND but exhibit taller, narrower peaks than observed experimentally.

**Figure 5:**
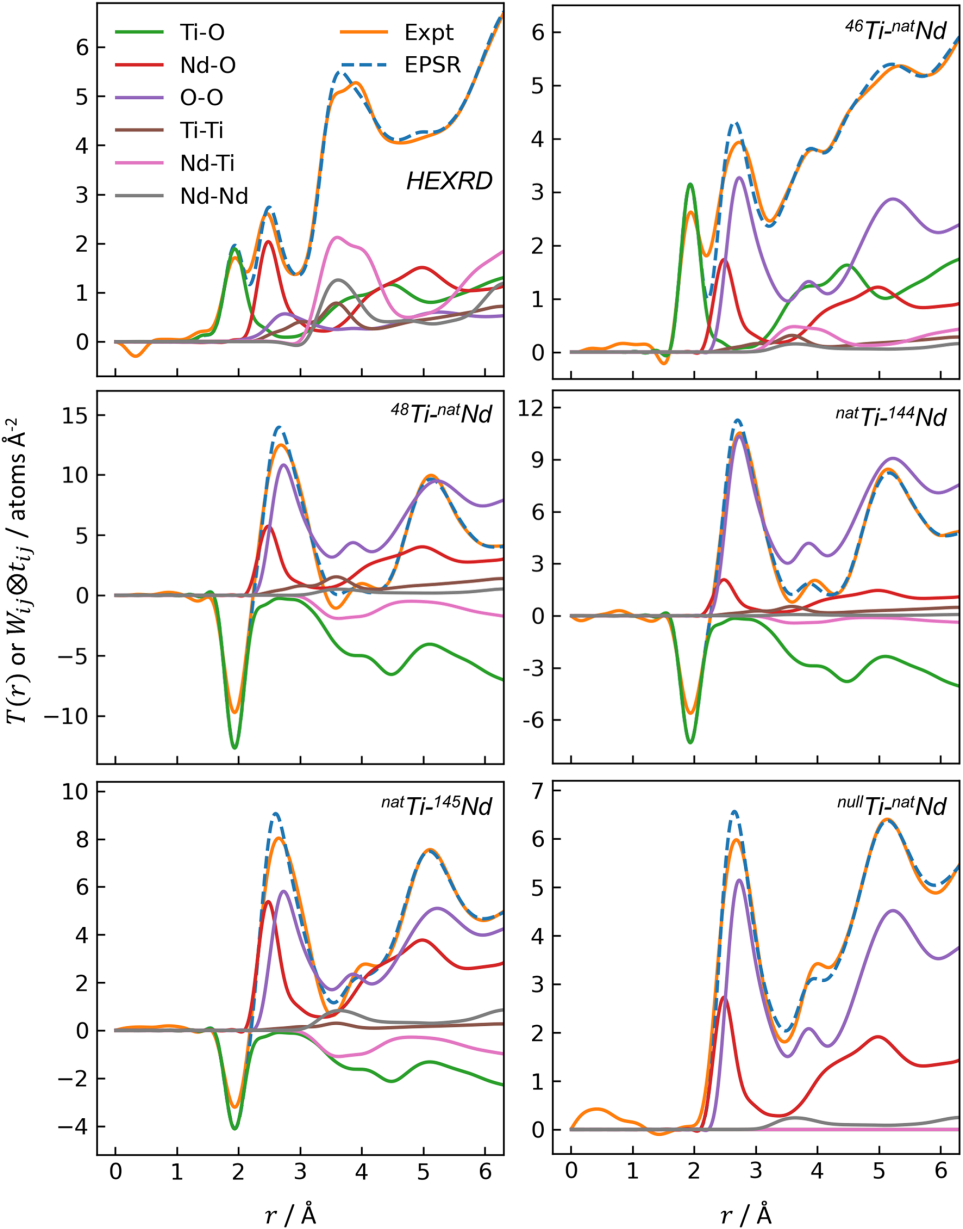
Comparison of the total PDFs (experimental in orange; EPSR in dashed blue) and their comprising weighted partial pair correlations for all measurements. All partials are from EPSR.

Figure 5 shows the contributions of the weighted partial pair correlations from EPSR to the total PDF for all measurements, which highlights the very different weighting of partials in different measurements. For example, for ^48^Ti-^nat^Nd ND, the Ti-O (green) and Nd-Ti (pink) correlations are negative due to the negative scattering length of ^48^Ti. In HEXRD, the O-O correlation (purple, $W_{OO}^{\prime }$ = 9.6%) is weak compared to cation-containing atomic pairs, while in ^48^Ti-^nat^Nd ND the O-O scattering is prominent ($W_{OO}^{\prime }$ = 39.9%). The HEXRD also illustrates the substantial overlap of the first peaks for Ti-O (green), Nd-O (red), and O-O (purple), which was one of the main motivations for this work, since analysis of a single total PDF is unreliable for obtaining coordination numbers when peaks overlap significantly.

#### Coordination environments

**Figure 6:**
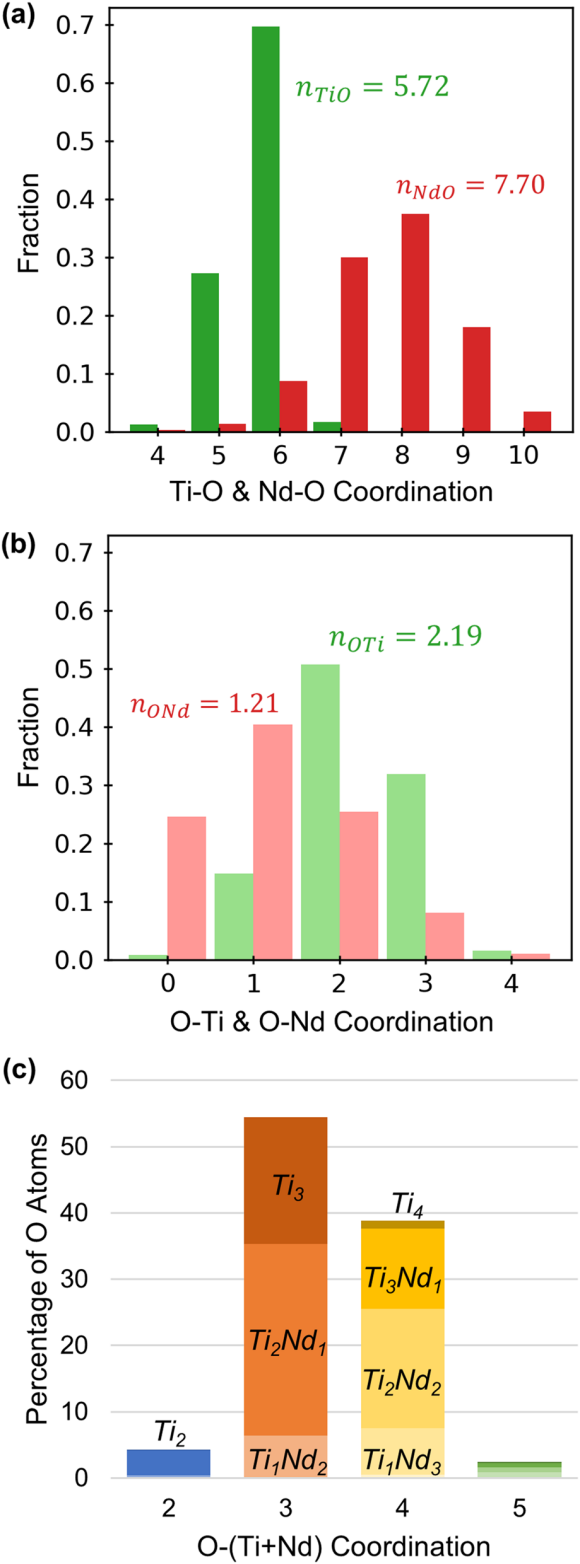
Coordination distributions from EPSR of (**a**) Ti-O (green) and Nd-O (red); (**b**) O-Ti and O-Nd; (**c**) combined O-(Ti + Nd).

The distributions of Ti-O and Nd-O coordination from EPSR are given in Fig. 6a, using cutoff distances of 2.6 and 3.25 Å, respectively. The Nd-O coordination is broadly, symmetrically distributed over the range of 5- to 10-coordinate, which agrees well with the Sm-O distribution reported by Maruyama et al*.* for a reverse Monte Carlo model of the compositionally similar Sm_4_Ti_9_O_24_ glass^11^. Ti-O species are predominantly 5-coordinate (26%) and 6-coordinate (73%). These values are more heavily weighted to 6-coordinate than those given by Arai et al*.*^5^ for La_4_Ti_9_O_24_ glass (65% 5-coordinate, 35% 6-coordinate) based on X-ray and neutron scattering measurements. Arai et al*.* separated the contributions of overlapping Ti-O and La-O peaks by fitting five Gaussian functions to an X-ray/neutron difference function (with O-O eliminated). Thus, the discrepancy in coordination numbers between Arai’s study and the work here likely stems from the uncertainty inherent to fitting of multiple, overlapping Gaussians. For Sm_4_Ti_9_O_24_, Maruyama et al*.* also reported a Ti-O distribution at lower coordination (16% 4-coordinate, 36% 5-coordinate, 37% 6-coordinate, 7% 7-coordinate)^11^ compared to the result here. The Sm_4_Ti_9_O_24_ model was refined using three ND measurements, so the EPSR model here, based on six independent measurements, should be more robust. Maruyama et al*.*’s result is also based on a much different glass density, for which the details of measurement were not reported. Nonetheless, there is broad agreement that the rare-earth titanate glass networks are largely composed of 5- and 6-fold Ti-O polyhedral units.

Mean Ti-O and Nd-O coordination numbers calculated from the EPSR distributions are $n_{TiO}$ = 5.72(6) and $n_{NdO}$ = 7.70(26), which are compared in Table 1 with the direct integration of ND first order differences, the compositionally nearest crystalline phase, Nd_4_Ti_9_O_24_, and prior studies on compositionally similar glasses. The EPSR and first order difference values differ by 5.2% for $n_{TiO}$ and 3.3% for $n_{NdO}$ (similar to the combined uncertainties of the two methods). Compared with crystalline Nd_4_Ti_9_O_24_ ($n_{TiO}$ = 6 and $n_{NdO}$ = 7.5), the 83TiO_2_-17Nd_2_O_3_ glass has lower coordination for Ti-O but higher coordination for Nd-O. The $n_{TiO}$ value aligns well with Alderman et al*.*’s^4^ Ti K-edge XANES powder transmission measurements on glasses of 83TiO_2_-17Nd_2_O_3_ ($n_{TiO}$ = 5.59(11)) and 83TiO_2_-17La_2_O_3_ ($n_{TiO}$ = 5.63(10)).

**Table 1:** Comparison of Ti-O and Nd-O coordination numbers from ND, EPSR, the nearest crystalline phase, and studies on compositionally similar rare-earth (RE) titanate glasses.

Method	$n_{TiO}$	$\overline{r}_{TiO}$(Å)	RE	$n_{REO}$	$\overline{r}_{REO}$(Å)
ND first order difference	5.43(15)	–	Nd	7.95(35)	–
EPSR	5.72(6)	1.984(11)	Nd	7.70(26)	2.598(22)
Nd_4_Ti_9_O_24_ crystal	6	1.974	Nd	7.5	2.475
XANES: 83TiO_2_-17Nd_2_O_3_ glass^4^	5.59(11)	–	Nd	–	–
XANES: 83TiO_2_-17La_2_O_3_ glass^4^	5.63(10)	–	La	–	–
HEXRD: 83TiO_2_-17La_2_O_3_ glass^4^	5.46(9)	1.942(2)	La	9.4(2)	2.531(4)
ND/HEXRD: La_4_Ti_9_O_24_ glass^5^	5.35	1.90	La	8.0	2.48
ND: Sm_4_Ti_9_O_24_ glass^11^	4.7	1.9	Sm	7.2	2.46

This study’s values for $n_{NdO}$ are lower than Alderman et al*.*’s^4^ $n_{LaO}$ = 9.4(2) for the lanthanum titanate glass, which is likely explained by the O-O partial contributing to their La-O peak fitting of the total PDF (see Fig. 5). For a similar lanthanum titanate glass, Arai et al*.*^5^ eliminated the O-O contribution using an HEXRD/ND difference, yielding $n_{LaO}$ = 8.0. In comparison, the $n_{NdO}$ found here is slightly lower, which is consistent with the smaller ionic radius of Nd^3+^ compared to La^3+^^3^.

Using the relationship,

5 $$n_{OTi} = \frac{{c_{Ti} }}{{c_{O} }}n_{TiO}$$

the average O-Ti coordination is 2.19 (or 2.08 using the ND difference function). A value of $n_{OTi}$ ~ 2 could correspond to a network of entirely bridging oxygen, similar to the topological ordering of many network glasses such as SiO_2_. Rare-earth titanate glasses generally exhibit 1.9 ≤ $n_{OTi}$ ≤ 2.1^4^, so the EPSR-predicted $n_{OTi}$ would suggest more O-Ti_3_ than previously reported. A significant fraction of these triclusters, a term first used for O linking three Al-O_4_ tetrahedra in aluminosilicate networks^32,33^, leads to topological hardening and lower glass-forming ability, similar to networks containing large fractions of Al_2_O_3_^34^ or TiO_2_^35^. In the cases of pure Al_2_O_3_ or TiO_2_, the large fraction of triclusters prevents glass formation via melt-quenching, though amorphous forms can be prepared by other means^36^. Here, O-Ti_3_ are referred to as triclusters, recognizing that they are linking three Ti-O_5_ or Ti-O_6_ polyhedra.

As shown in Fig. 6b, the O-Ti coordination distribution contains 15% O-Ti_1_, 51% O-Ti_2_, and 32% O-Ti_3_. This large fraction of triclusters is possibly an overestimation by the model since EPSR, like other Monte Carlo methods, yields maximally entropic structures that are consistent with the experimental data. In this case, some disproportionation of 2(O-Ti_2_) into O-Ti_1_ and O-Ti_3_ would increase the model’s entropy while maintaining the same mean coordination. Nonetheless, the tricluster fraction is much lower than that estimated for amorphous TiO_2_ and Al_2_O_3_, which cannot practically be melt quenched to form glasses. Molecular dynamics models for melt-quenched TiO_2_ and Al_2_O_3_ glasses indicate TiO_2_ containing 75% O-Ti_3_ and 25% O-Ti_2_^35^ and Al_2_O_3_ containing 82% O-Al_3_ and 7% O-Al_2_^37^. That is, much higher tricluster fractions than in the rare-earth titanate glasses.

The O-Nd coordination distribution (Fig. 6b) contains 25% O atoms not bonded to Nd, with 40% O-Nd_1_ and 25% O-Nd_2_. Additional insights can be gleaned from the combined distribution for O-(Ti + Nd) coordination, Fig. 6c, which shows a variety of O bonding environments. The majority of O (93%) are bonded to 3 or 4 cations. Of the nonbridging O-Ti_1_Nd_x_, roughly half are bonded with 2 Nd atoms and half bond with 3 Nd. Of the bridging O-Ti_2_Nd_x_, 7% are not bonded to Nd, 57% bond with 1 Nd, and 36% bond with 2 Nd. Of the O-Ti_3_Nd_x_ triclusters, 63% are bonded to no Nd and 37% bond with 1 Nd atom.

**Figure 7:**
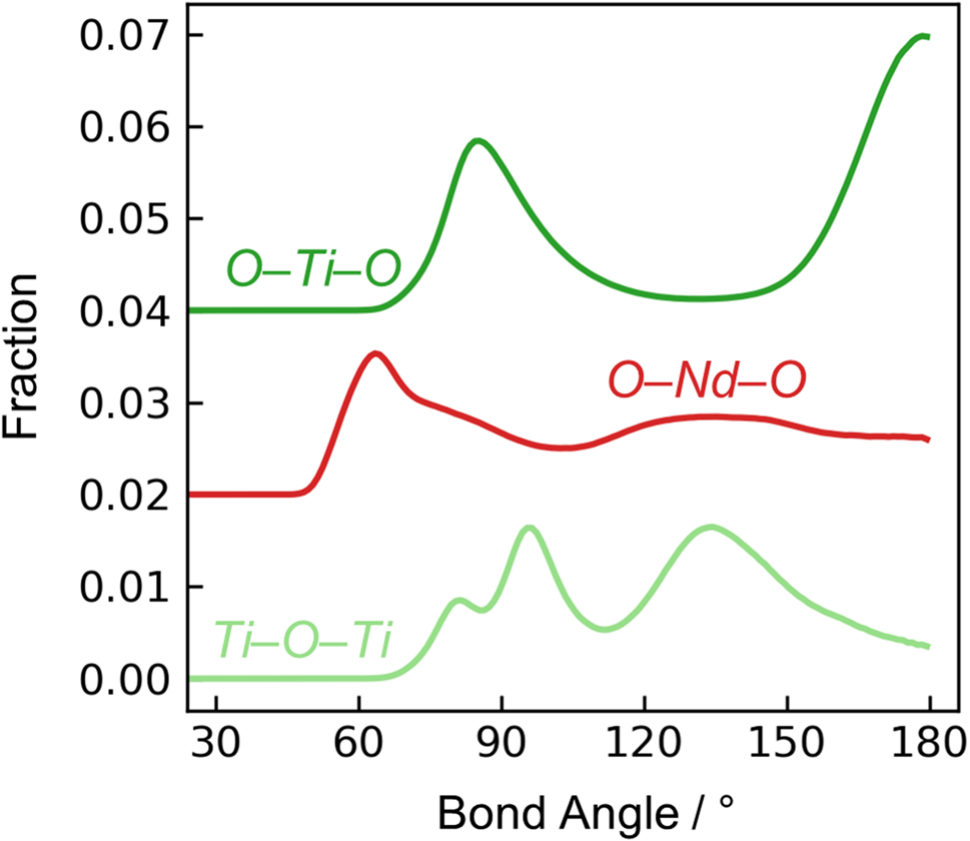
Bond angle distributions from EPSR. Curves are vertically offset for clarity.

Bond angle distributions obtained from EPSR, Fig. 7, are consistent with the polyhedral configurations expected from the coordination number distributions (Fig. 6). (For Fig. 7, bond angle distributions are shown after division by the $sin\left( \theta \right)$ dependence that would occur for a random distribution of angles^38^.) The O-Ti-O bond angles are bimodally distributed around 90° and 180°, consistent with 5- or 6-coordinate arrangements. The broad distributions of angles indicate a variety of distorted polyhedra, rather than the ideal trigonal bipyramidal, square pyramidal, and octahedral configurations. The lack of a peak at 120°, expected for trigonal bipyramidal, is further evidence that the Ti-O_5_ are highly distorted or perhaps predominantly square pyramidal. When O-Ti-O bond angles are calculated separately for Ti-O_4_, Ti-O_5_, and Ti-O_6_ species, the distributions are similar except that Ti-O_6_ exhibits a larger relative fraction of bond angles between 160–180° (Fig. S5). The O-Nd-O bond angle distribution has a peak around 65° but is otherwise broadly distributed across all angles up to 180°, again consistent with a variety of distorted polyhedra with a range of Nd-O coordination (Fig. 6a, red bars). The modal coordination of Nd-O_8_, assuming O atoms are equally spaced at corners of a cube around the Nd, would appear at 70.5°, which is roughly in agreement with the peak near 65° in Fig. 7. The Ti-O-Ti bond angle distribution is reflective of the glass network topology: a broad peak around 135° represents corner-sharing Ti-O_x_ polyhedra, and a double-peak near 90° represents edge-sharing polyhedra. The splitting of the peak at 90° may be a result of the many distinct O-(Ti + Nd) bonding environments (Fig. 6c).

#### Bond distances

Mean bond distances in EPSR were calculated from the running atomic coordination, with cutoff distances of 2.6 Å for Ti-O and 3.25 Å for Nd-O. For comparison, the Ti-O and Nd-O bond distances were analyzed for crystalline Nd_4_Ti_9_O_24_^19^ using GSAS-II^39^ and are compared in Table 1. For the glass, $\overline{r}_{TiO}$ = 1.984(11) Å, in close agreement with the crystalline Nd_4_Ti_9_O_24_ average (1.974 Å). The slightly larger Ti-O mean bond length in the glass may appear contradictory, given that Ti-O coordination is lower in the glass compared to the crystal. However, this is consistent with the distortion theorem for bond valence models, which explains why distorted polyhedra (e.g., in a glass) have larger mean bond lengths compared to regular polyhedra (e.g., in crystalline phases) of the same coordination^40,41^. The mean bond length $\overline{r}_{OTi}$ was calculated separately for O-Ti_1_, O-Ti_2_, and O-Ti_3_ species—1.948(5), 1.961(2), and 2.015(3) Å—indicating longer bonds for higher coordination O environments, as expected. The glass Nd-O mean bond length, $\overline{r}_{NdO}$ = 2.598(22) Å, is slightly longer than the Nd_4_Ti_9_O_24_ crystal (2.475 Å), which is consistent with the higher Nd-O coordination in the glass and an elongation of the mean anticipated by the distortion theorem. The values for $\overline{r}_{TiO}$ and $\overline{r}_{NdO}$ here are both larger than Alderman et al*.*’s^4^ 83TiO_2_-17La_2_O_3_ glass ($\overline{r}_{TiO}$ = 1.942(2) Å, $\overline{r}_{LaO}$ = 2.531 Å), which is likely due to subtle structural differences arising from the different rare-earth cations^4^.

Bond valence theory can provide an additional consistency check of bond distances and coordination numbers obtained from diffraction experiments^42,43^. Using Ti-O as an example, the bond valence equation for identical length bonds relates cation valence, $v$, to coordination and bond distance:

6 $$v_{Ti} = {\text{exp}}\left( {\frac{{R_{TiO} - r}}{b}} \right)n_{TiO}$$

where $R_{TiO}$ and $b$ are the bond valence parameters^44^. However, for asymmetric distributions of bond distances, as seen here for Ti-O and Nd-O, Eq. 6 cannot be directly applied. Instead, following Hannon^43^, the running bond valence sum can be expressed in terms of the partial pair correlation, $t_{TiO}$:

7 $$V_{Ti} \left( r \right) = \mathop \smallint \limits_{0}^{r} c_{O} t_{TiO} \left( {r^{\prime } } \right)\exp \left( {\frac{{R_{TiO} - r^{\prime } }}{b}} \right)r^{\prime } dr^{\prime }$$

This integral is similar to that used for determining the coordination number (Eqn. S14 in the SI) except it includes the exponential bond valence weighting term. When integrating over the entire Ti-O peak, $V_{Ti}$ should match the formal charge for Ti. Applying Eq. 7 to the EPSR-predicted Ti-O correlation with a cutoff of 2.6 Å gives a bond valence sum of 3.93, close to that expected for Ti^4+^. For Nd-O with a cutoff of 3.25 Å, the bond valence sum gives 2.50, which is 17% smaller than that expected for Nd^3+^. As a comparison, the Nd_4_Ti_9_O_24_ crystalline phase has an Nd bond valence sum of 2.83, also considerably smaller than the formal charge of 3+ . Still, the smaller value of 2.50 for the glass suggests the presence of some longer Nd-O bonds that contribute to satisfying the valence requirements; however, these longer bonds overlap with the second Nd-O coordination shell and thus cannot be isolated for inclusion in the bond-valence calculation.

#### Glass network

**Figure 8:**
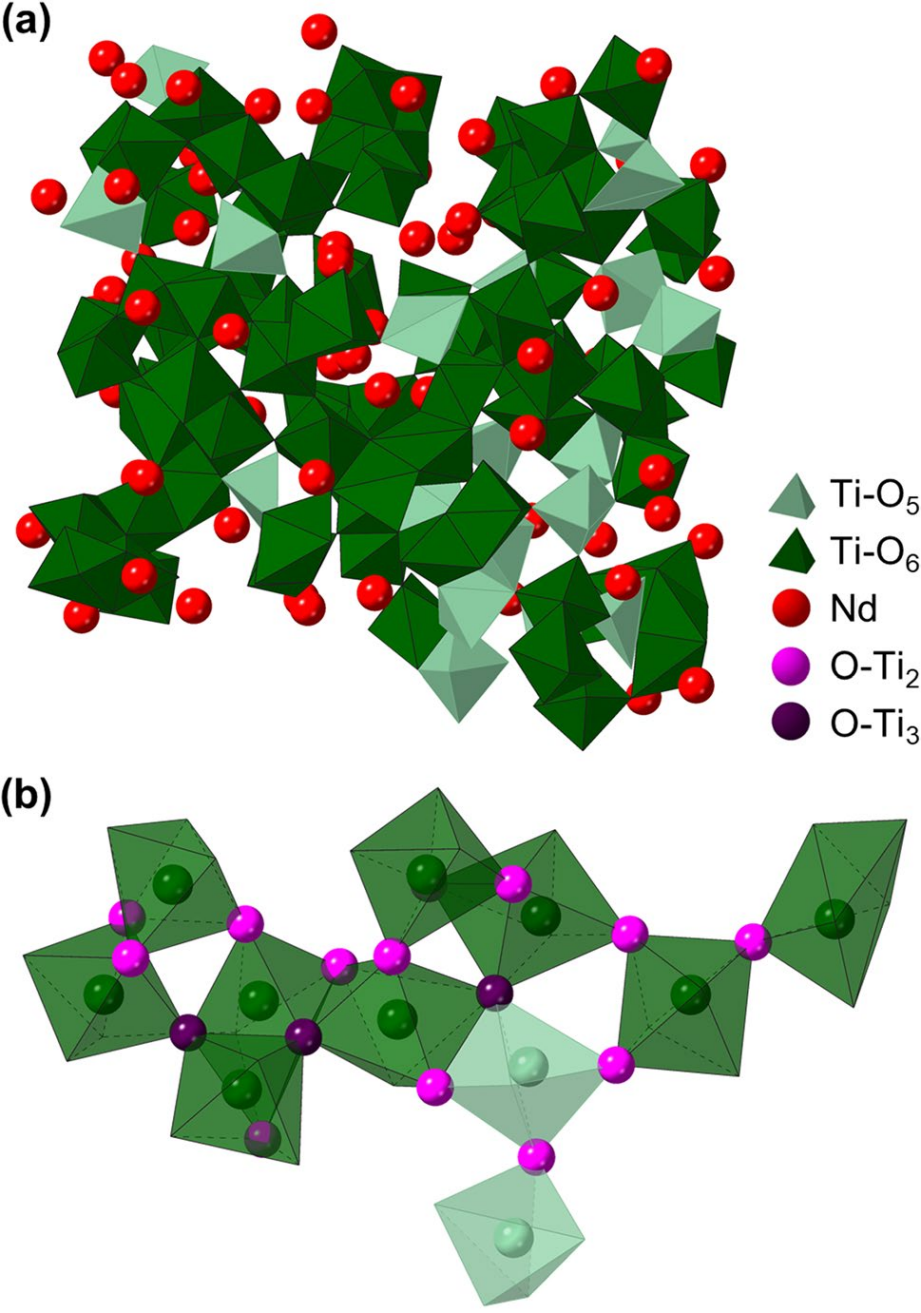
Selected visualizations of EPSR model. (**a**) Network structure, illustrating Ti-O_5_ and Ti-O_6_ polyhedra and Nd atoms. (**b**) Ti-O_x_ polyhedra are connected via bridging oxygen (O-Ti_2_) and triclusters (O-Ti_3_), predominantly through corner- and edge-sharing. For clarity, nonbridging O (O-Ti_1_) are not shown.

The glass network, illustrated in Fig. 8, comprises a mixture of Ti-O_5_ and Ti-O_6_ polyhedra with Nd atoms acting akin to network modifiers. The Ti-O_x_ polyhedra are connected via bridging O atoms (O-Ti_2_, magenta in Fig. 8b) and O triclusters (O-Ti_3_, dark purple in Fig. 8b). Bridging O are present where polyhedra share corners or edges, while triclusters can correspond to instances of edge- or face-sharing. The network connectivity is characterized by 71% corner-sharing, 23% edge-sharing, and 6% face-sharing. The crystal Nd_4_Ti_9_O_24_, in contrast, has a much smaller fraction of corner-sharing, 38%, with 62% edge-sharing and no face-sharing.

This network, comprising mostly octahedral Ti-O_6_, is highly unusual compared to most oxide glasses prepared by melt quenching of liquids at ambient pressure. In comparison to canonical glass-forming systems such as SiO_2_, which comprises Si in only tetrahedral coordination with O, the near absence of Ti-O_4_ tetrahedra here (1.7%, see Fig. 6a) is particularly striking. Previous reports of oxide glass networks built of cations in higher (> 4) coordination have mostly focused on high pressure systems using in situ measurements. For example, GeO_2_ exhibits Ge-O_x_ average coordination of 4 below 5 GPa, ~ 5 between 6–10 GPa, and ~ 6 at 15 GPa^45^. However, this octahedral glass is unstable: it is not retained upon return to ambient pressure.

**Figure 9:**
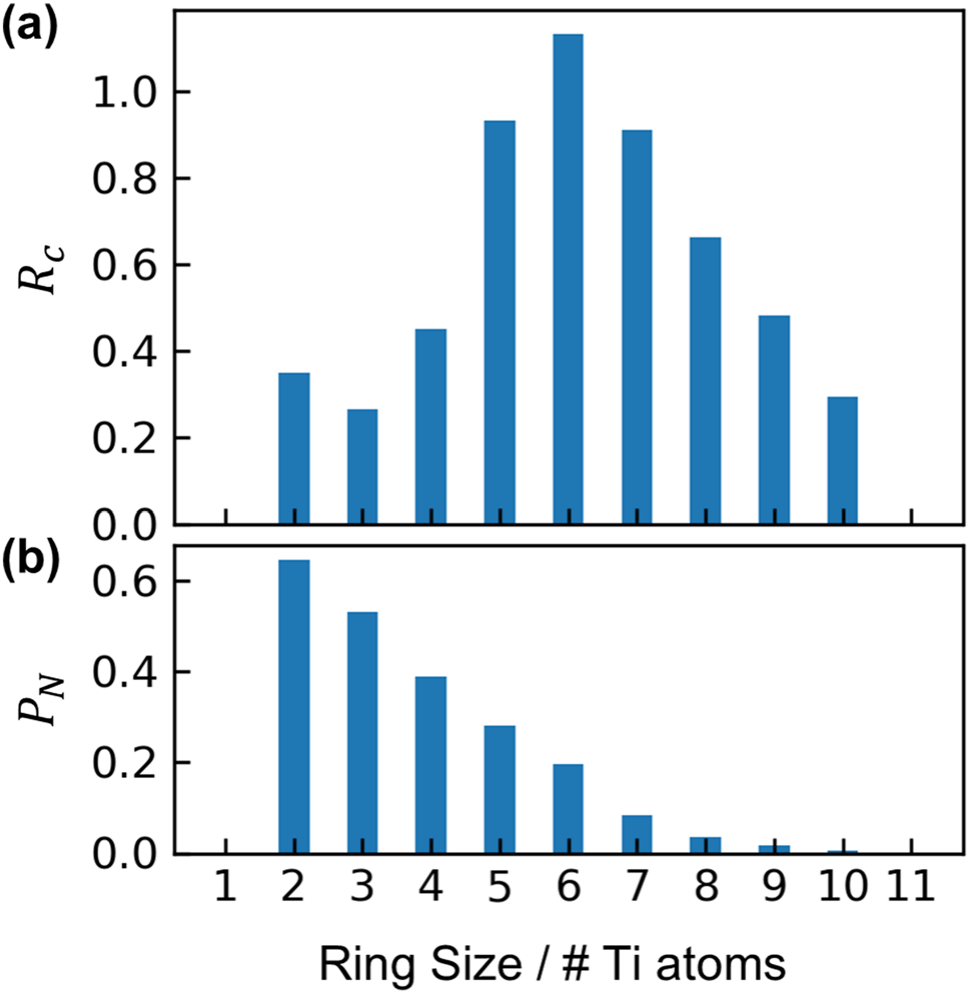
(**a**) Distribution of -O-Ti-O- ring sizes in EPSR model, and (**b**) fraction of atoms participating in rings of a given size.

Figure 8 also illustrates the existence of -O-Ti-O- rings of various sizes. The ring size distribution was calculated using King’s criterion^46^ and is provided in Fig. 9a in terms of $R_{c}$, the number of rings of a given size, normalized by the number of Ti + O atoms in the simulated volume. $R_{c}$ is distributed roughly symmetrically around a modal ring size of $N$ = 6 Ti atoms, with sizes ranging from 2 to 10 Ti atoms. However, in analyzing ring statistics it is important to consider the proportion of atoms participating in rings of a given size, which is given by the statistic $P_{N}$. This is especially true for networks with significant edge-sharing, such as this neodymium titanate glass, because King’s criterion yields large ring counts for big rings that are nearly identical except for tracing one of two possible paths across an edge-share (i.e., using one of the two O-Ti_2_ that define a single edge-share)^47^. For example, a ring containing 3 edge-sharing connections would be counted as 2^3^ = 8 unique rings. While these rings are indeed unique, this effect magnifies exponentially with ring size and can result in a large $R_{c}$ for big rings, even if they are not characteristic of the network in general. As shown in Fig. 9b, $P_{N}$ decreases monotonically with ring size, which can be understood in the context of significant (23%) edge-sharing. Thus, while $N$ = 6 rings are the distribution’s mode, only 20% of atoms participate in these rings. Rings containing only 2 Ti atoms represent pairs of edge-sharing Ti-O_x_ polyhedra, which are highly representative of the network ($P_{N}$ = 64%).

In general, the distribution of ring sizes is located across smaller rings than those found in the corner-sharing tetrahedral networks of canonical glass formers such as SiO_2_, which has a modal ring size of $N$ = 7 Si atoms and ~ 28% of atoms participating in rings of that size^47^.

## Conclusions

Neodymium titanate (83TiO_2_-17Nd_2_O_3_) was melted and vitrified via aerodynamic levitation and laser heating, and its atomic structure was investigated with a combination of X-ray diffraction (HEXRD), neutron diffraction (ND) with isotope substitution of Ti and Nd, and empirical potential structure refinement (EPSR). The atomic partial pair correlations that overlap in the total pair distribution function (PDF) were isolated using neutron difference functions and the EPSR structural model, which describes well the six diffraction measurements (5 ND + 1 HEXRD). The Ti-O and Nd-O coordination numbers are lower and higher, respectively, than those in the compositionally similar crystal phase, Nd_4_Ti_9_O_24_. The Ti-O coordination, $n_{TiO}$ = 5.72(6) from EPSR or 5.43(15) from ND first order difference, is in good agreement with past Ti X-ray absorption near-edge structure measurements and HEXRD/ND on compositionally similar lanthanum titanate glasses. The Nd-O coordination, $n_{NdO}$ = 7.70(26) from EPSR or 7.95(35) from ND first order difference, improves upon past HEXRD/ND studies of 83TiO_2_-17La_2_O_3_, which were not able to fully distinguish the La-O peak from overlapping contributions of the Ti-O and O-O partials. Bond angles for O-Ti-O and O-Nd-O are consistent with polyhedral configurations expected from the coordination number distributions and indicate a variety of highly distorted polyhedra. The titanate glass network is built of Ti-O_5_ and Ti-O_6_ polyhedra connected via corner- and edge-sharing, leading to O coordination environments that are 15% O-Ti_1_ (nonbridging), 51% O-Ti_2_ (bridging), and 32% O-Ti_3_ (tricluster). Statistical analysis of -O-Ti-O- rings in the network yields a modal ring size of 6 Ti atoms, though the presence of significant edge-sharing results in large counts for big rings ($N \ge$ 6) that are not representative for most atoms of network. For example, 64% of atoms participate in rings containing 2 Ti atoms, making these representative of the network, whereas only 20% of atoms participate in rings with 6 Ti atoms. This glass network, consisting predominantly of octahedral Ti-O_6_, is atypical for melt-quenching of liquids at ambient pressure, as octahedral glasses have generally been observed only in situ during high-pressure studies.

## Methods

### Sample preparation

Glasses of 83TiO_2_-17Nd_2_O_3_ were prepared for six samples with different isotopic substitutions: ^46^Ti-^nat^Nd, ^48^Ti-^nat^Nd, ^nat^Ti-^144^Nd, ^nat^Ti-^145^Nd, ^null^Ti-^nat^Nd, and ^nat^Ti-^nat^Nd. Natural abundance and isotopically enriched powders of rutile TiO_2_ and Nd_2_O_3_ (Isoflex; purity and enrichment given in Table S3) were calcined in Pt crucibles at 600 °C for 3 h to remove adsorbed water and then weighed and mixed in the desired ratio, accounting for isotopic masses. The ^null^Ti-^nat^Nd sample used a mixture of ^46^TiO_2_ and ^nat^TiO_2_ powders in the correct proportion for the average Ti coherent neutron scattering length to be zero. Powder mixtures were mixed thoroughly and then melted in a copper hearth with a 10.6 μm CO_2_ laser to obtain fused spheroids, which were ground in a mortar and pestle and remelted to ensure macrohomogeneity. Spheroids were then levitated on an oxygen gas stream in a conical nozzle levitator^15^ and laser heated to 2300–2350 °C for 30 s to ensure complete melting (Nd_2_O_3_ $T_{m}$ = 2233°C^16^). Temperature measurements were made with an optical pyrometer (λ = 0.9 μm) and corrected using a sample spectral emissivity of 0.92^48^.

### Energy dispersive X-ray spectroscopy

One bead of ^nat^Ti-^nat^Nd glass was mounted in epoxy, mechanically ground, and polished to a final step with 1 μm diamond suspension. The resulting smooth cross-section was used for glass composition measurements via energy dispersive X-ray spectroscopy (EDS; Hitachi SU8030) with quantitative standardization for Ti and Nd.

### Helium pycnometry

The volume of each ND sample was measured using helium pycnometry (Anton Paar Ultrapyc 5000 Micro) in a 0.25 cm^3^ cell. At least 16 measurements were made per sample and then averaged, excluding outliers (usually the first one or two measurements). Density was calculated by dividing the sample mass (± 0.1 mg) by the measured volume.

### High energy X-ray diffraction

HEXRD measurements were collected at the Advanced Photon Source, Sector 6-ID-D, at Argonne National Laboratory (Lemont, IL, USA). Glass beads were loaded into thin-walled (10 μm thick) 3 mm diameter silica capillaries, and the diffracted intensity of 99.98 keV X-rays was measured in transmission geometry using a Varex 4343CT area detector and a sample-to-detector distance of ~ 350 mm, yielding a range of momentum transfers 0.6 < $Q$ < 27 Å^−1^. The X-ray beam was roughly 250 μm wide $\times$ 100 μm tall, so only a small portion of one bead was measured at a time. Measurements across several locations per bead were reproducible.

For PDF analysis, the measured raw scattering data must be corrected and reduced to obtain the structure factor^17,49^. To begin, the area detector signal was azimuthally integrated and corrected for flat field effects and X-ray polarization using Fit2D software^50^. Corrections were then applied for oblique incidence and detector attenuation^51^, and GudrunX software^52^ was used for background subtraction (i.e., scattering from an empty capillary and air) and corrections for X-ray fluorescence, sample attenuation and multiple scattering. The resulting total scattering intensity from the sample, defined as $I_{X} \left( Q \right)$, is related to the total scattering differential cross section:

8 $$\left( {\frac{d\sigma }{{d\Omega }}} \right)_{X} = \frac{{I_{X} \left( Q \right)}}{\rho V}$$

where $\rho$ is the sample’s atomic number density and $V$ is the probed volume. The total differential cross section can be expressed as a sum of distinct, self, and inelastic scattering:

9 $$\left( {\frac{d\sigma }{{d\Omega }}} \right)_{X} = \left( {\frac{d\sigma }{{d\Omega }}} \right)_{X,dist} + \left( {\frac{d\sigma }{{d\Omega }}} \right)_{X,self} + \left( {\frac{d\sigma }{{d\Omega }}} \right)_{X,inel}$$

The normalized total scattering structure factor, $S_{X} \left( Q \right)$, can then be expressed as:

10 $$S_{X} \left( Q \right) - 1 = \frac{{\left( {\frac{d\sigma }{{d\Omega }}} \right)_{X,dist} }}{{<f\left( Q \right)>^{2} }} = \frac{{\left( {\frac{d\sigma }{{d\Omega }}} \right)_{X} - \left( {\frac{d\sigma }{{d\Omega }}} \right)_{X,self} - \left( {\frac{d\sigma }{{d\Omega }}} \right)_{s,inel} }}{{<f\left( Q \right)>^{2} }}$$

where the denominator is the square of the composition-averaged, $Q$-dependent X-ray atomic form factors^53^, $f\left( Q \right)$. The self-scattering cross section is equal to the composition-average of the square of the form factors:

11 $$\left( {\frac{d\sigma }{{d\Omega }}} \right)_{X,self} = <f\left( Q \right)^{2}>$$

and the inelastic cross section corresponds to $Q$-dependent Compton scattering^54^. Structure factors were obtained from GudrunX. Measurement uncertainty was taken to be the square root of $I_{X} \left( Q \right)$.

Since the aforementioned corrections are inevitably imperfect, diffraction structure factors almost always contain some residual, long-wavelength background in $Q$-space, which was removed using the “top hat” convolution^18^ in Gudrun. The top hat convolution reduces the magnitude of nonphysical oscillations at small real-space distances in the PDF ($r$ < 1.2 Å), but otherwise does not alter the PDF (see Fig. S6 in the SI). The differential PDF, $D\left( r \right)$, was obtained by the sine Fourier transform of the interference function, $Q\left( {S\left( Q \right) - 1} \right)$:

12 $$D\left( r \right) = \frac{2}{\pi }\mathop \smallint \limits_{0}^{{Q_{max} }} Q\left( {S\left( Q \right) - 1} \right)M\left( Q \right)\sin \left( {Qr} \right)dQ$$

where $M\left( Q \right)$ is a Lorch modification function^55^, and a $Q_{max}$ of 18 Å^−1^ was used. The total PDF was calculated as:

13 $$T\left( r \right) = D\left( r \right) + 4\pi \rho r$$

14 $$T\left( r \right) = \mathop \sum \limits_{i,j \ge i} W_{ij} \otimes t_{ij}$$

The total PDF is a summation of the atomic partial pair correlations, $t_{ij}$, convolved by their normalized Faber-Ziman weighting factors^20^, $W_{ij}$ (Eq. 2).

HEXRD was measured on all six isotopically unique samples to confirm compositional uniformity. Glass density was estimated from HEXRD by fitting Gaussian functions with NXFit software^56^ to the first two peaks in the PDF and then minimizing the sum-square difference between $D\left( r \right)$ without those peaks and $- 4\pi \rho r$ over the range of $r$ = 0–1.4 Å (Fig. S2).

### Neutron diffraction

ND measurements were collected at the Nanoscale-Ordered Materials Diffractometer^57^ at the Spallation Neutron Source, Oak Ridge National Laboratory (Oak Ridge, TN, USA). Glass beads were again loaded into thin-walled, 3 mm diameter silica capillaries, and the resulting vertical columns of stacked beads ranged 8–12 mm tall. (Bead counts and masses are given in Table S1). At the measurement position, the neutron beam shape was roughly Gaussian with a FWHM of 6 mm.

As with HEXRD, the measured raw scattering data from ND must be reduced and normalized to obtain the structure factor, in preparation for PDF analysis. Full details of the ND data reduction and normalization are in the SI. The normalized total scattering structure factor from ND, $S_{N} \left( Q \right)$, is defined as:

15 $$S_{N} \left( Q \right) - 1 = \frac{{\left( {\frac{d\sigma }{{d\Omega }}} \right)_{N,dist} }}{{<b>^{2} }} = \frac{{\left( {\frac{d\sigma }{{d\Omega }}} \right)_{N} - \left( {\frac{d\sigma }{{d\Omega }}} \right)_{N,self} - \left( {\frac{d\sigma }{{d\Omega }}} \right)_{N,inel} - \left( {\frac{d\sigma }{{d\Omega }}} \right)_{N,mag} }}{{<b>^{2} }}$$

where $b$ are the $Q$-independent coherent neutron scattering lengths, taken from Sears^22^. For ND, the inelastic cross section is handled by a Placzek correction^58^, and a differential scattering cross section is included for Nd^3+^ paramagnetic scattering^59,60^.

Measurement uncertainty was taken to be the square root of the number of scattered neutrons detected. PDFs were obtained using Eqns. 12 and 13 with a Lorch modification function and $Q_{max}$ of 18 Å^−1^.

### Empirical potential structure refinement

A structural model for 83TiO_2_-17Nd_2_O_3_ glass was simulated with EPSR^21,31^, using the ND and HEXRD data as constraints. EPSR is a Monte Carlo based atomistic modeling technique, in which conventional atomic pair potentials are supplemented with an empirical potential derived from the disagreement between the modeled structure and experimental measurements. In this fashion, the empirical potential drives the simulation to favor Monte Carlo moves that improve model-experiment agreement.

Atomic pair interactions were implemented as a combination of Lennard–Jones and pseudo-Coulomb potentials (parameters given in Table S4 in the SI). A simulation box of edge length 30 Å was initialized with 2004 atoms randomly arranged throughout, which was equilibrated at 1000 °C and then at 27 °C. The empirical potential was then activated, first at a magnitude of 69 kJ mol^−1^ (10% of the system energy) and then at 103 kJ mol^−1^ (15% of the system energy). The structural model was then analyzed to obtain atomic partial pair correlations, coordination number distributions for Ti-O, Nd-O, O-Ti, and O-Nd, and bond angle distributions. Each segment of the simulation—equilibration, empirical potential, and analysis—was run for at least 10,000 iterations.

The combined coordination distribution for O-(Ti + Nd) was obtained using the R.I.N.G.S. code^47^ to analyze every 10th configuration over 9,000 EPSR iterations. Ring statistics were calculated for the same 900 configurations with R.I.N.G.S., using King’s criterion for shortest path and a maximum search depth (i.e., ring size) of 11 Ti atoms. The fractions of corner-, edge-, and face-sharing connections between Ti-O_x_ polyhedra were calculated by tabulating all Ti-Ti nearest neighbor pairs and then counting the number of O atoms bonded to each Ti atom in a given Ti-Ti neighbor pair (i.e., within the Ti-O bond cutoff distance of 2.6 Å). Corner-, edge-, and face-sharing conditions correspond to Ti-Ti nearest neighbors that mutually bond to 1, 2, or 3 shared O atoms, respectively. Visualizations of the glass network (Fig. 8) were rendered with CrystalMaker 10.6 software.

## Supplementary Information

Supplementary Information 1.

Supplementary Information 2.

Supplementary Information 3.

Supplementary Information 4.

Supplementary Information 5.

Supplementary Information 6.

Supplementary Information 7.

## Data Availability

All data are available upon reasonable request from the corresponding authors. Structure factor data are provided in the SI for the six diffraction measurements.
