# Supplementary material for: Octahedral oxide glass network in ambient pressure neodymium titanate
Source: Sci Rep. 2022 May 18;12:8258. doi: 10.1038/s41598-022-12342-x (PMC9117677; doi:10.1038/s41598-022-12342-x)
Supplement: Supplementary file 7 — Supplementary Information 7. [file 41598_2022_12342_MOESM7_ESM.pdf]

Supplementary Information for:

Octahedral oxide glass network in ambient pressure neodymium titanate

Stephen K. Wilke, Oliver L.G. Alderman, Chris J. Benmore, Jörg Neuefeind, Richard Weber

Data Reduction and Normalization of Neutron Diffraction

ND was performed at the Nanoscale-Ordered Materials Diffractometer of the Spallation Neutron Source, a time-of-flight instrument. Scattering was measured in an argon-filled reentrant well for each sample, an empty silica capillary, and for a vanadium standard. For $83TiO_2$-$17Nd_2O_3$, the sample scattering (after subtraction of the empty capillary and argon background), I_s, can be related to the total scattering differential cross section, which is the summation of the per-atom distinct, self, inelastic, and magnetic scattering differential cross sections:

$$\frac{I_s}{\rho_s V_s} = \left(\frac{d\sigma}{d\Omega}\right)_s = \left(\frac{d\sigma}{d\Omega}\right)_{s,dist} + \left(\frac{d\sigma}{d\Omega}\right)_{s,self} + \left(\frac{d\sigma}{d\Omega}\right)_{s,inel} + \left(\frac{d\sigma}{d\Omega}\right)_{s,mag} \tag{S1}$$

where ρ_s is the atomic number density and V_s is the sample volume. (Effects of sample absorption were checked and confirmed to be negligible, so all expressions here are given without absorption corrections.) The vanadium scattering, I_v, can be expressed similarly while noting the absence of magnetic scattering and that incoherent (i.e., self) scattering is the dominant term:

$$\frac{I_v}{\rho_v V_v} = \left(\frac{d\sigma}{d\Omega}\right)_v \approx \left(\frac{d\sigma}{d\Omega}\right)_{v,self} = {b_{v,inc}}^2 \tag{S2}$$

where $b_{v,inc}$ is vanadium's incoherent scattering length.

The goal of data reduction is to isolate the sample's distinct scattering, from which the total scattering structure factor can be obtained (e.g., Eqn. 15 in the main text). By defining the ratio of sample-to-vanadium scattering,

$$I_R \equiv \frac{I_s}{I_v} \tag{S3}$$

and combining with Eqns. S1-S2, the sample distinct scattering is given by:

$$\left(\frac{d\sigma}{d\Omega}\right)_{s,dist} = \frac{I_R \rho_v V_v {b_{v,inc}}^2}{\rho_s V_s} - \left(\frac{d\sigma}{d\Omega}\right)_{s,self} - \left(\frac{d\sigma}{d\Omega}\right)_{s,inel} - \left(\frac{d\sigma}{d\Omega}\right)_{s,mag} \tag{S4}$$

Self scattering is equal to the composition-averaged square of the incoherent scattering lengths:

$$\left(\frac{d\sigma}{d\Omega}\right)_{s,self} = \langle {b_{s,inc}}^2 \rangle \tag{S5}$$

Inelastic scattering is handled by a Placzek correction[1], and the paramagnetic scattering is given in terms of a magnetic form factor for Nd^{3+} [2,3]. However, given the small sample sizes (e.g., I_R < 0.08), it was

difficult to fully correct the weak sample scattering using these expressions. Instead, an empirical fit using a pseudo-Voigt function, I_{base}, is used to account for the combination of self, inelastic, and magnetic scattering. Eqn. S4 can then be simplified to:

$$\left(\frac{d\sigma}{d\Omega}\right)_{s,dist} = A(I_R - I_{base}) \tag{S6}$$

where A is a lumped, sample-dependent (Q-independent) constant. The experimentally measured I_R and empirically fitted I_{base} are shown for the five ND samples in Fig. S7.

Finally, the normalized total scattering structure factor, $S_N(Q)$, is given by:

$$S_N(Q) - 1 = \frac{\left(\frac{d\sigma}{d\Omega}\right)_{s,dist}}{\langle b_s \rangle^2} = \frac{A}{\langle b_s \rangle^2}(I_R - I_{base}) \tag{S7}$$

where $\langle b_s \rangle$ is the composition-average coherent scattering length of the sample. The neutron differential PDF is then obtained using Eqn. 12, which combined with Eqn. S7 yields:

$$D(r) = \alpha \int_0^{Q_{max}} Q(I_R - I_{base}) M(Q)\, sin(Qr)\, dQ \tag{S8}$$

where $\alpha = 2A/\pi\langle b_s \rangle^2$ is a sample-dependent constant. As described in the main text for HEXRD analysis, $M(Q)$ is a Lorch modification function and the Gudrun top hat convolution was used with $S_N(Q)$ to remove residual, long-wavelength background in Q-space. ND total PDFs were calculated according to Eqn. 13.

The normalization of ND data is now equivalent to determining the appropriate value of α for each of the five ND samples. This normalization was optimized by using the theoretical limiting behavior of $T(r) = 0$ at low-r (i.e., at real-space distances shorter than any bonds). Even with the top hat convolution, some residual background in Q-space results in oscillations at low- r, so the most robust determination of α is obtained by using as large a range of r as possible. Toward this goal, for each sample, the $T(r)$ obtained from HEXRD and ND were weighted so as to eliminate the first peak (from the t_{TiO} partial) in an HEXRD/ND difference function. Because the HEXRD weighting factors are Q-dependent, this difference must be performed in Q-space prior to the Fourier transformation. Beginning with Eqns. 12-13 and dividing by the Ti-O weighting factor, W_{TiO}, of each measurement, the difference function is defined as:

$$\begin{aligned} \Delta_{X-N} = &\left[\frac{2}{\pi}\int_0^{Q_{max}} Q\,\frac{(S_X(Q)-1)}{W_{TiO,X}(Q)} M(Q)\sin(Qr)\,dQ + \frac{4\pi\rho r}{W_{TiO,X}(Q=0)}\right] \\ &- \left[\frac{2}{\pi}\int_0^{Q_{max}} Q\,\frac{(S_N(Q)-1)}{W_{TiO,N}} M(Q)\sin(Qr)\,dQ + \frac{4\pi\rho r}{W_{TiO,N}}\right] \end{aligned} \tag{S9}$$

$$\begin{aligned} \Delta_{X-N} = &\frac{2}{\pi}\int_0^{Q_{max}} Q\,\frac{(S_X(Q)-1)}{W_{TiO,X}(Q)} M(Q)\sin(Qr)\,dQ + 4\pi\rho r\left(\frac{1}{W_{TiO,X}(Q=0)} - \frac{1}{W_{TiO,N}}\right) \\ &- \frac{D_N(r)}{W_{TiO,N}} \end{aligned} \tag{S10}$$

This expression for Δ_{X-N} in principle should be zero at low-r. With the Ti-O peak now eliminated, the value of α (contained in the expression for D_N in Eqn. S10) was determined by minimizing the sum-square of Δ_{X-N} over r = 0-2.0 Å.

Neutron Diffraction First Order Difference Functions

For the purpose of explanation, the Ti first order difference will be described, though the same methodology applies for the Nd first order difference. The total PDF for a neutron diffraction (ND) sample can be expressed as a summation of the atomic partial pair correlations, t_{ij}, as given in Eqn. 14. Because the weighting factors, W_{ij}, are Q-independent for ND, Eqns. 2 and 14 can be rearranged to give:

$$\langle b\rangle^2 T(r) = \sum_{i,j\geq i}(2-\delta_{ij})c_i c_j b_i b_j t_{ij} \tag{S11}$$

For a pair of samples that are identical in composition except for substitution of Ti isotopes, all terms in the summation of Eqn. S11 not containing i or j = Ti will be equivalent between samples. Thus, the difference function has only atomic-pairs containing Ti:

$$\begin{aligned}\langle b\rangle_1^2 T_1 - \langle b\rangle_2^2 T_2 = c_{Ti}^2\left(b_{Ti,1}^2 - b_{Ti,2}^2\right)t_{TiTi} + 2c_{Ti}c_{Nd}\left(b_{Ti,1} - b_{Ti,2}\right)b_{Nd}t_{TiNd} \\ +2c_{Ti}c_O\left(b_{Ti,1} - b_{Ti,2}\right)b_O t_{TiO}\end{aligned} \tag{S12}$$

where "1" and "2" subscripts represent the ^{46}Ti-^{nat}Nd and ^{48}Ti-^{nat}Nd samples, respectively. Since we are most interested in using the difference function to investigate Ti-O coordination, the pertinent function is defined as:

$$\begin{aligned}\Delta_{Ti}(r) &= \frac{\langle b\rangle_1^2 T_1 - \langle b\rangle_2^2 T_2}{2c_{Ti}c_O\left(b_{Ti,1} - b_{Ti,2}\right)b_O} \\ &= t_{TiO} + \frac{c_{Ti}\left(b_{Ti,1}^2 - b_{Ti,2}^2\right)t_{TiTi} + 2c_{Nd}\left(b_{Ti,1} - b_{Ti,2}\right)b_{Nd}t_{TiNd}}{2c_O\left(b_{Ti,1} - b_{Ti,2}\right)b_O}\end{aligned} \tag{S13}$$

for which the first peak will correspond to the unweighted partial t_{TiO}.

For a given atomic partial pair correlation, t_{ij}, the average coordination number is given by:

$$n_{ij} = \int_{r_1}^{r_2} c_j t_{ij}(r) r dr \tag{S14}$$

where the integral bounds are selected to bracket the peak in the PDF. Since the t_{TiTi} and t_{TiNd} correlations do not overlap with the first peak of t_{TiO}, the function Δ_{Ti} can be used to integrate the first peak and obtain the Ti-O coordination:

$$n_{TiO} = \int_{1.50}^{2.61} c_O \Delta_{Ti}(r) r dr \tag{S15}$$

The same process was used to obtain the function $\Delta_{Nd}(r)$ from the ^{nat}Ti-^{144}Nd and ^{nat}Ti-^{145}Nd samples, and n_{NdO} was determined according to Eqn. S15 and integrating over r = 2.00-3.40 Å. Fig. 4 provides a plot of $\Delta_{Ti}(r)$ and $\Delta_{Nd}(r)$.

Estimating Uncertainty in Atomic Coordination Numbers

Five sources of uncertainty were evaluated during the calculation of atomic coordination numbers: diffraction measurement uncertainty, samples' composition uncertainty, ^{145}Nd scattering length, sample density, and ND normalization.

The measurement uncertainty arises from counting statistics (i.e., Poisson noise), which was propagated through the data reduction to obtain $S(Q)$ and through the Fourier transform to obtain the PDFs[4,5]. The contribution of measurement uncertainty was small relative to effects of density and ND normalization, and thus was not considered in the final analysis.

Uncertainty in the samples' chemical compositions leads to uncertainty in the composition-average scattering lengths: $\langle b \rangle$ for ND or $\langle f \rangle$ for HEXRD. Based on the energy dispersive spectroscopy reported in the main text, the samples' compositions were off-nominal by 1.2 mol. % Nd_2O_3. Assuming this value provides an upper bound for the uncertainty among different samples, the compositional variation leads to 1-2% changes in $\langle b \rangle^2$ or $\langle f \rangle^2$, which contributes less uncertainty to the final analysis than the effects of density and ND normalization. Thus, compositional uncertainty was not considered in the final analysis.

The coherent neutron scattering length for ^{145}Nd has never been directly measured, and the value provided by Sears (b = 14(2) fm)[6] was deduced from the scattering lengths known for ^{nat}Nd and some of the Nd isotopes at the time, assuming that ^{143}Nd and ^{145}Nd would have the same scattering length. To probe the effect of this uncertainty, ND difference functions and EPSR were carried out using b = 14 ± 2 fm for ^{145}Nd. The uncertainty propagated to the results (e.g., coordination numbers) was smaller than that arising from density and ND normalization, so it was not considered further. Given the scattering length uncertainty of 2/14 ≈ 14%, this at first seems surprising. However, because Nd has the lowest atomic concentration in the glass, the Nd-containing weighting factors are already quite small, which explains the small effect of ^{145}Nd scattering length uncertainty.

HEXRD was identified as the most reliable method for estimating density, as discussed in the main text. Density and its uncertainty were defined as the weighted mean and standard deviation of values extracted from HEXRD measurements of the six samples. This uncertainty was propagated through the analysis (e.g., ND difference functions and EPSR) to obtain the corresponding uncertainty in atomic coordination numbers.

For the ND normalization procedure described earlier in the SI, the HEXRD/ND difference function (Eqn. S10) was calculated with each of the six HEXRD measurements, yielding six values for the normalization constant, α, for each sample. The effect of ND normalization uncertainty on atomic coordination numbers was determined by considering the extreme cases of different possible α values. For example, the Ti ND difference function was first calculated using the mean values of α for ^{46}Ti-^{nat}Nd and ^{48}Ti-^{nat}Nd (resulting in n_{TiO} = 5.43). Then the ND difference was recalculated using the maximum α for ^{46}Ti-^{nat}Nd and the minimum α for ^{48}Ti-^{nat}Nd (resulting in n_{TiO} = 5.42), and recalculated once more using the minimum α for ^{46}Ti-^{nat}Nd and the maximum α for ^{48}Ti-^{nat}Nd (n_{TiO} = 5.48). This approach yielded the full range of coordination numbers that might result from the ND normalization uncertainty.

ND normalization depends on the sample density, so the overall uncertainty in coordination number was defined as the maximum of the two contributions (density and ND normalization). For n_{TiO}, the density effect was larger (uncertainty of ± 0.15 in ND difference calculation), and for n_{NdO} the ND normalization effect was larger (uncertainty of ± 0.35 in ND difference calculation).

Atomic-Pair Partial Structure Factors from Weights Matrix Inversion

The solution to Eqn. 3 is obtained by inverting the weights matrix, which must be completed for each discrete Q value of the structure factors because the weighting factors for HEXRD are Q-dependent. To provide an example of the relative magnitudes of terms in the inverted matrix, the solution to Eqn. 3 for Q = 0 is:

$$\begin{bmatrix} s_{TiTi} \\ s_{TiNd} \\ s_{TiO} \\ s_{NdNd} \\ s_{NdO} \\ s_{OO} \end{bmatrix} = \begin{bmatrix} 10.93 & 2.70 & 0 & 0 & -12.63 & 0 \\ -13.40 & 5.17 & -6.30 & -12.56 & 23.88 & 4.21 \\ 4.83 & -1.49 & 1.31 & 2.61 & -5.38 & -0.87 \\ 18.39 & -16.37 & 19.82 & 29.30 & -50.14 & 0 \\ -7.10 & 4.79 & -7.08 & -7.04 & 16.48 & 0.95 \\ 2.15 & -1.28 & 2.09 & 1.66 & -3.22 & -0.40 \end{bmatrix} \begin{bmatrix} S_1 \\ S_2 \\ S_3 \\ S_4 \\ S_5 \\ S_6 \end{bmatrix} \quad \text{(S16)}$$

The partial structure factors (Fig. S3) and partial PDFs (Fig. S4) were calculated using the fully Q-dependent solution to Eqn. 3. The partial s_{TiTi} also corresponds to the Ti second order neutron difference function, which is a linear combination of the ^{46}Ti-^{nat}Nd (S_1), ^{48}Ti-^{nat}Nd (S_2), and ^{null}Ti-^{nat}Nd (S_5) ND structure factors.

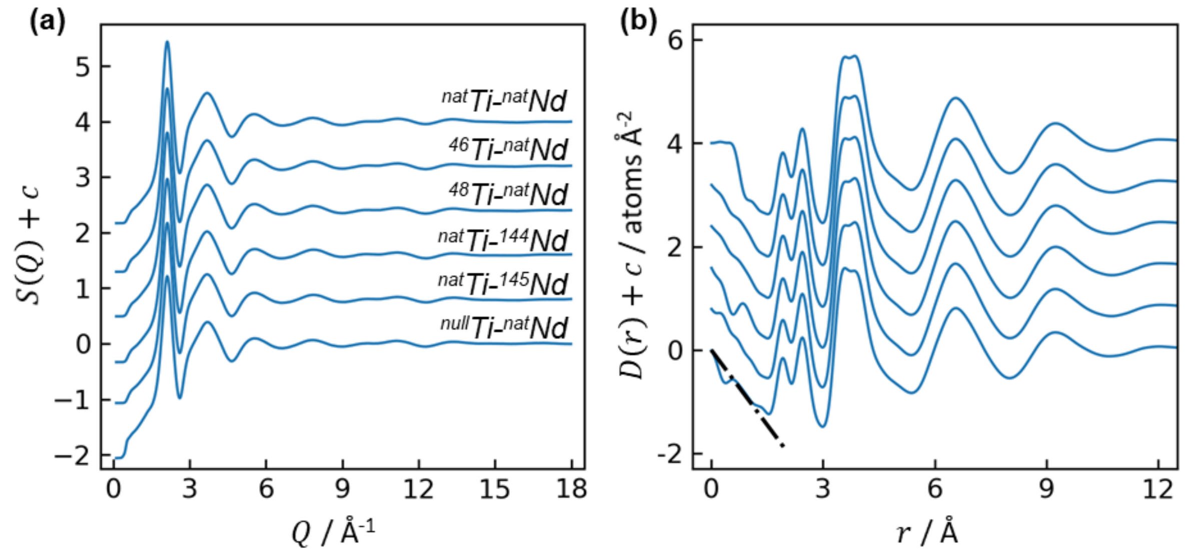

Figure S1. HEXRD (a) structure factors and (b) differential PDFs for all six samples of $83TiO_2$-$17Nd_2O_3$. Curves are vertically offset for ease of viewing. Dashed black line indicates the initial slope given by $-4\pi\rho$.

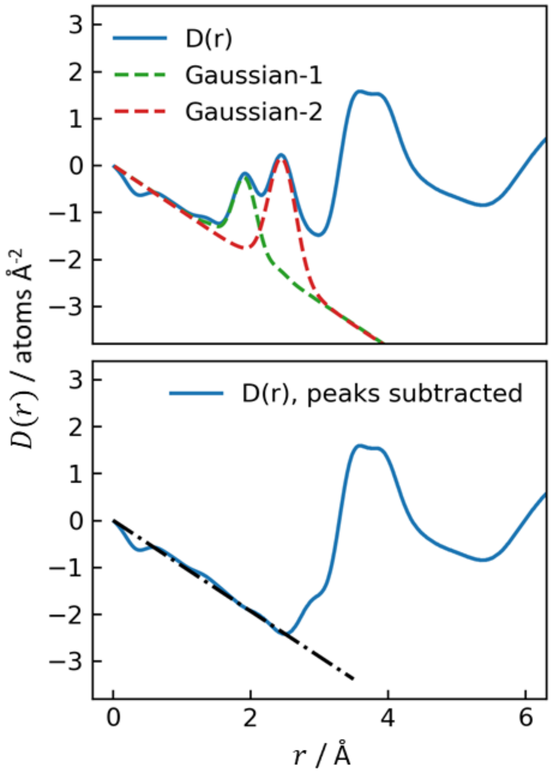

Figure S2. Determination of sample density from one sample's HEXRD differential PDF. Top: Gaussian functions were fit to the first two peaks in $D(r)$. Bottom: after subtracting the fitted peaks, the sample density, ρ, was determined by minimizing the sum-square difference between $D(r)$ and $-4\pi\rho r$ over 0-1.4 Å.

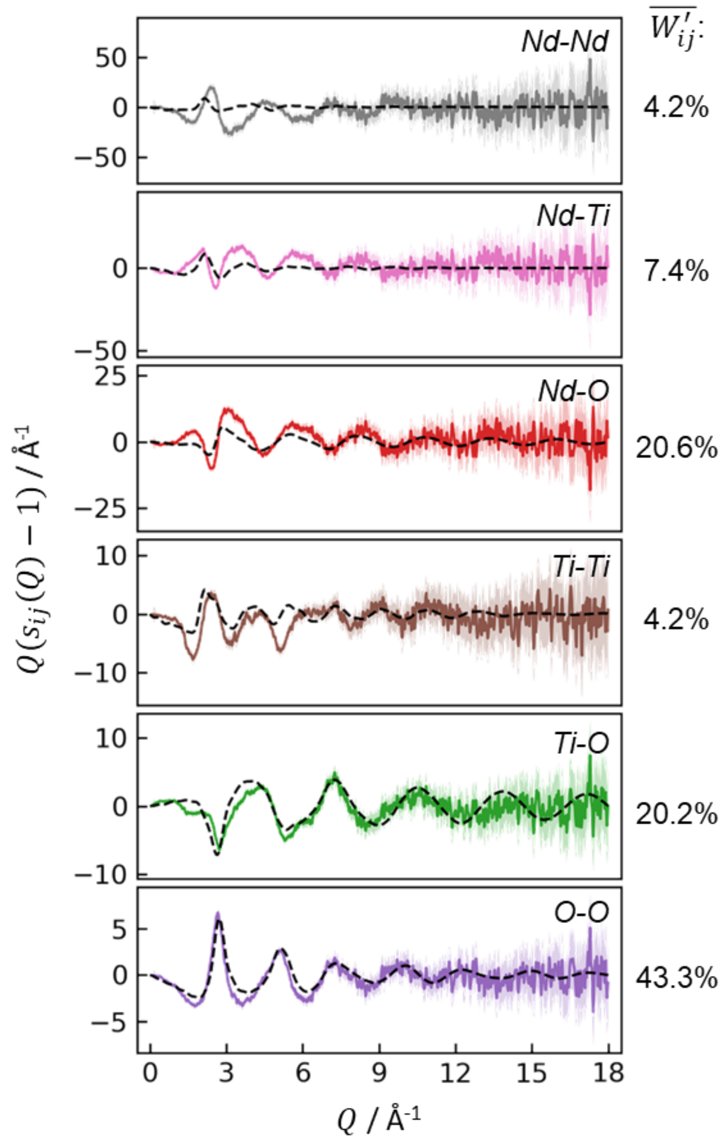

Figure S3. Partial structure factors obtained from the solution to Eqn. 3 and Eqn. S16. Uncertainty arising from the experimental measurements is shown in light shading. Dashed black curves show partials from EPSR. Mean values are given for the modified weighting factors, W'_{ij}.

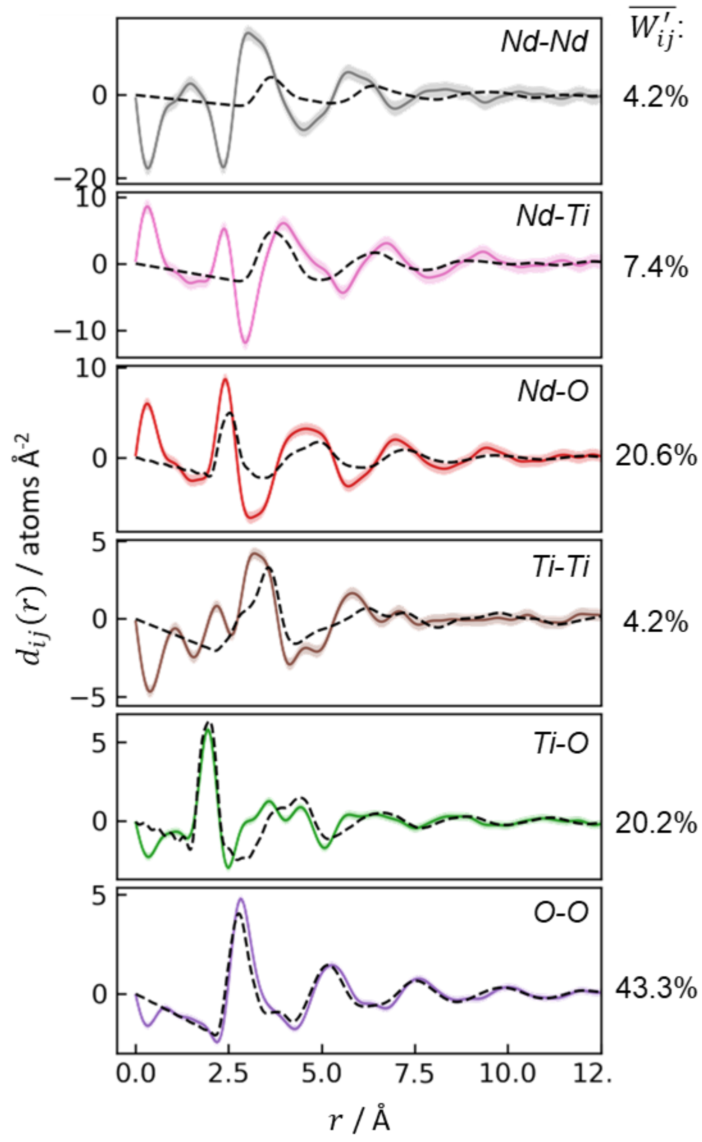

Figure S4. Partial PDFs obtained from the solution to Eqn. 3 and Eqn. S16, i.e., after the Fourier transform of partial structure factors shown in Fig. S3 with a Q_{max} of 12 Å^{-1}. Uncertainty arising from the experimental measurements is shown in light shading. Dashed black curves show partials from EPSR (also using a Q_{max} of 12 Å^{-1}). Mean values are given for the modified weighting factors, W'_{ij}.

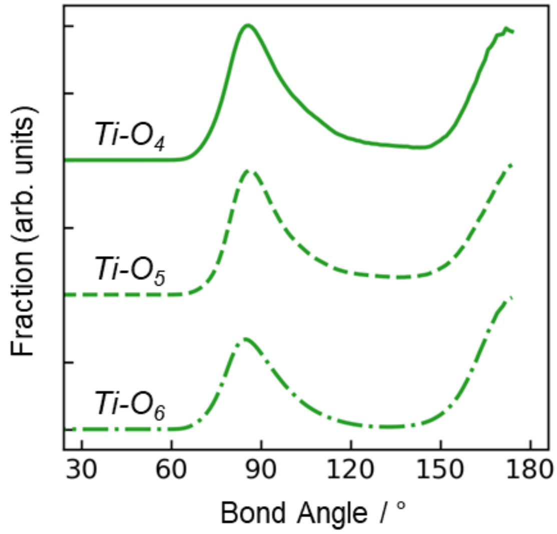

Figure S5. Distributions of O-Ti-O bond angles from EPSR, calculated separately for Ti-O_4, Ti-O_5, and Ti-O_6 species. Curves are vertically offset for clarity.

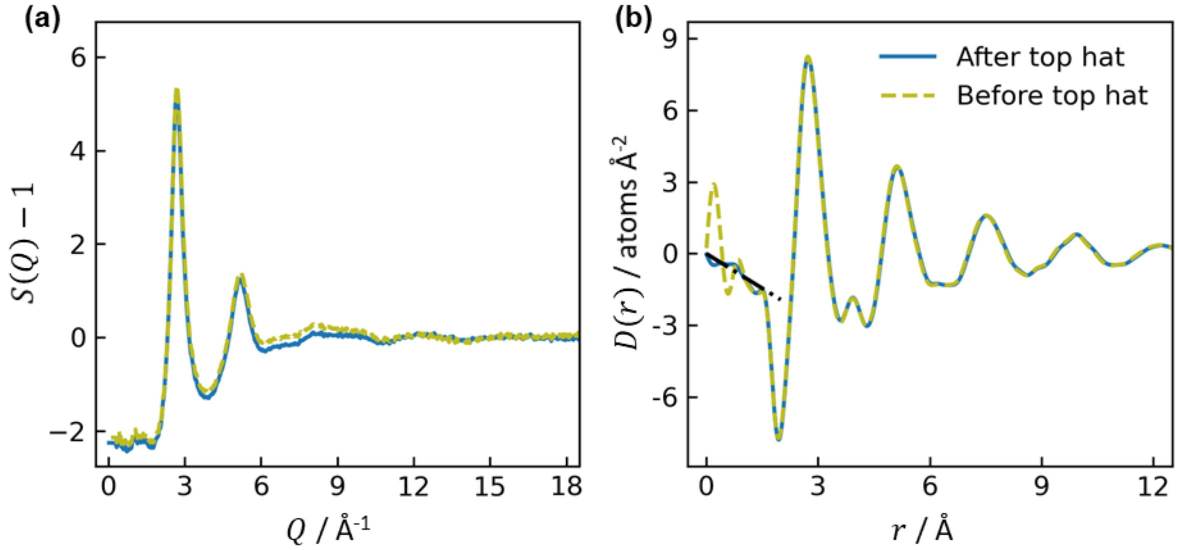

Figure S6. Comparison of (a) the structure factor and (b) the differential PDF for the ^{nat}Ti-^{144}Nd sample before and after the top hat convolution[7] in Gudrun[8]. The top hat convolution alters only the nonphysical oscillations at low-r in the PDF. Dashed black line indicates the initial slope given by $-4\pi\rho$.

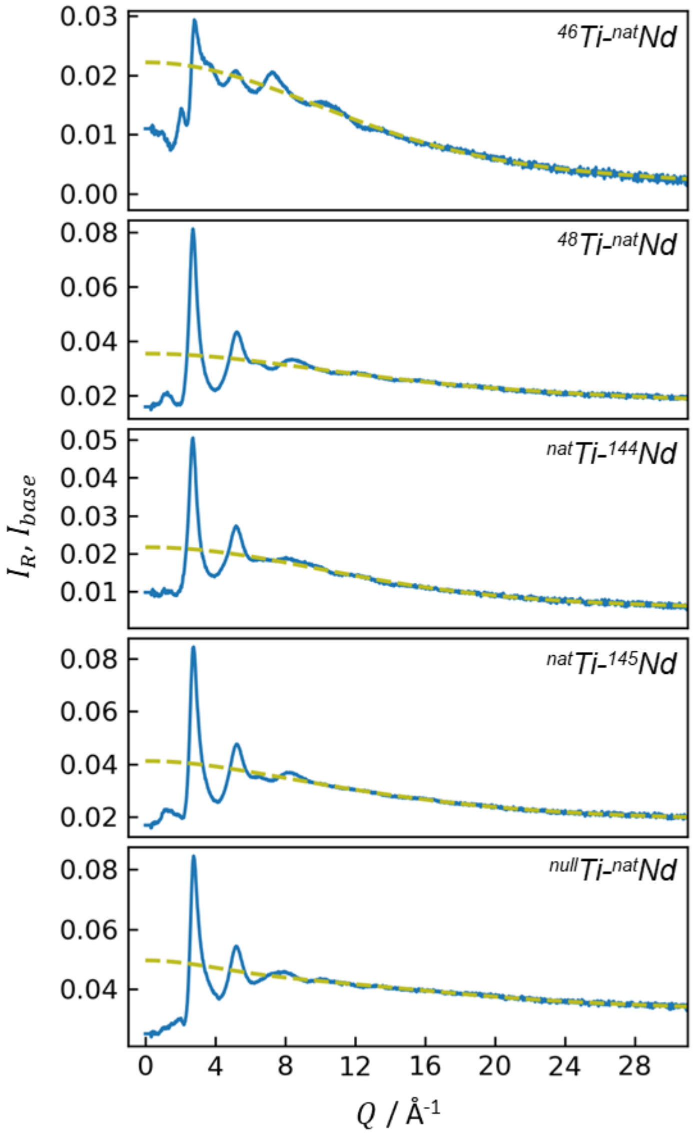

Figure S7. The ratio of ND sample scattering to scattering of the vanadium standard (blue curve, I_R in Eqn. S6), and the empirically fitted baseline (dashed green, I_{base} in Eqn. S6) that includes self, inelastic, and Nd^{3+} paramagnetic scattering.

Table S1. Samples' number of glass beads, total mass, volume measured by gas pycnometry, and density calculated from pycnometry. Volume uncertainties each indicate the standard deviation of at least 16 measurements. Systematic measurement errors arising from the very small sample volumes likely explain the variation in calculated densities across samples.

	^{46}Ti-^{nat}Nd	^{48}Ti-^{nat}Nd	^{nat}Ti-^{144}Nd	^{nat}Ti-^{145}Nd	^{null}Ti-^{nat}Nd	Mean
Beads (#)	7	8	6	9	10	
Mass (mg)	67.4(1)	82.1(1)	62.5(1)	93.4(1)	114.3(1)	
Volume (mm^3)	13.98(71)	16.65(71)	15.20(78)	22.52(66)	24.22(75)	
Density (g cm^{-3})	4.82(25)	4.93(21)	4.11(21)	4.15(12)	4.72(15)	4.55(39)

Table S2. Scattering lengths and normalized Faber-Ziman weighting factors, W_{ij}, for the $83TiO_2$-$17Nd_2O_3$ glasses with different isotopic substitution. Percentage weighting factors, W'_{ij}, are defined in Eqn. 4.

	^{46}Ti-^{nat}Nd ND	^{48}Ti-^{nat}Nd ND	^{nat}Ti-^{144}Nd ND	^{nat}Ti-^{145}Nd ND	^{null}Ti-^{nat}Nd ND	^{nat}Ti-^{nat}Nd HEXRD	Mean
b_{Ti} (fm)	4.669	-5.692	-3.438	-3.438	0.000	22*	
b_{Nd} (fm)	7.690	7.690	2.914	13.434	7.690	60*	
b_{O} (fm)	5.803	5.803	5.803	5.803	5.803	8*	
$\langle b\rangle^2$ (fm^2)	32.64	9.85	10.32	18.35	20.73	281.3*	
W_{TiTi}	0.0412	0.2031	0.0707	0.0398	0.0000	0.1062	
W_{TiNd}	0.0557	-0.2248	-0.0491	-0.1274	0.0000	0.2374	
W_{TiO}	0.2680	-1.0828	-0.6242	-0.3511	0.0000	0.2020	
W_{NdNd}	0.0188	0.0622	0.0085	0.1019	0.0296	0.1326	
W_{NdO}	0.1808	0.5992	0.2168	0.5620	0.2847	0.2257	
W_{OO}	0.4355	1.4430	1.3773	0.7747	0.6857	0.0960	
W'_{TiTi}	4.1	5.6	3.0	2.0	0.0	10.6	4.2
W'_{TiNd}	5.6	6.2	2.1	6.5	0.0	23.7	7.4
W'_{TiO}	26.8	30.0	26.6	17.9	0.0	20.2	20.2
W'_{NdNd}	1.9	1.7	0.4	5.2	3.0	13.3	4.2
W'_{NdO}	18.1	16.6	9.2	28.7	28.5	22.6	20.6
W'_{OO}	43.5	39.9	58.7	39.6	68.6	9.6	43.3

* The natural abundance sample was used for HEXRD, for which the Q-dependent atomic form factors, $f(Q)$, are used instead of the coherent neutron scattering length, b, to calculate the weighting factors. Form factors and weighting factors for HEXRD listed here are for Q = 0 Å^{-1}.

Atomic fractional concentrations are c_{Nd} = 0.1018, c_{Ti} = 0.2485, c_{O} = 0.6497. Scattering lengths for pure isotopes are taken from Sears[6], and the elemental scattering lengths shown here are calculated using certificates of analysis for the isotope-enriched powders.

Table S3. Chemical purity and isotopic enrichment of starting powders for glass synthesis.

**Powder**	**Purity (%)**	**Enrichment (%)**
$^{nat}TiO_2$	99.99	--
$^{46}TiO_2$	98.05	97.0
$^{48}TiO_2$	98.98	96.2
$^{nat}Nd_2O_3$	99.999	--
$^{144}Nd_2O_3$	99.99	98.7
$^{145}Nd_2O_3$	> 98	91.7

Table S4. Parameters for starting atomic potentials in EPSR.

Ion	Charge (e)	ε (kJ mol^{-1})	σ (Å)	Mass (amu)	Ref.
Ti^{4+}	+2	2.23	1.6	47.88	[9]
Nd^{3+}	+1.5	1.5	2.8	144.24	--
O^{2-}	-1	0.92	3.0	16.00	[9]

Structure factor data for the five ND and one X-ray measurements are provided as text file attachments in the Supplementary Information:

SoQ_ND_46Ti_natNd.dat
SoQ_ND_48Ti_natNd.dat
SoQ_ND_natTi_144Nd.dat
SoQ_ND_natTi_145Nd.dat
SoQ_ND_nullTi_natNd.dat
SoQ_Xray.dat

References (for Supplementary Information)

1. Placzek, G. The Scattering of Neutrons by Systems of Heavy Nuclei. *Phys. Rev.* **86**, 377–388 (1952).
2. Koehler, W. C. & Wollan, E. O. Paramagnetic scattering of neutrons by rare earth oxides. *Phys. Rev.* **92**, 1380–1386 (1953).
3. Wezka, K. *et al.* Structure of praseodymium and neodymium gallate glasses. *J. Non. Cryst. Solids* **357**, 2511–2515 (2011).
4. Skinner, L. B. *et al.* Benchmark oxygen-oxygen pair-distribution function of ambient water from x-ray diffraction measurements with a wide Q-range. *J. Chem. Phys.* **138**, 074506 (2013).
5. Weitkamp, T., Neuefeind, J., Fischer, H. E. & Zeidler, M. D. Hydrogen bonding in liquid methanol at ambient conditions and at high pressure. *Mol. Phys.* **98**, 125–134 (2000).
6. Sears, V. F. Neutron scattering lengths and cross sections. *Neutron News* **3**, 26–37 (1992).
7. Soper, A. K. Inelasticity corrections for time-of-flight and fixed wavelength neutron diffraction experiments. *Mol. Phys.* **107**, 1667–1684 (2009).
8. Soper, A. K. & Barney, E. R. Extracting the pair distribution function from white-beam X-ray total scattering data. *J. Appl. Crystallogr.* **44**, 714–726 (2011).
9. Alderman, O. L. G., Benmore, C. J., Neuefeind, J., Tamalonis, A. & Weber, R. Molten barium titanate: A high-pressure liquid silicate analogue. *J. Phys. Condens. Matter* **31**, 20LT01 (2019).
